# Development of a Public-Domain Measure of Two-Dimensional Rotation Ability and Preliminary Evidence for Discriminant Validity among Occupations

**DOI:** 10.3390/jintelligence11100191

**Published:** 2023-10-03

**Authors:** Kendall A. Mather, David M. Condon

**Affiliations:** Psychology Department, University of Oregon, Eugene, OR 97403, USA; dcondon@uoregon.edu

**Keywords:** spatial ability, scale development, two-dimensional rotation, STEM, cognitive ability

## Abstract

Despite their known influence in science, technology, engineering, and mathematics (STEM) fields, spatial abilities remain an underassessed aspect of cognition, particularly in educational settings. One explanation could be a lack of affordable, valid instruments for measuring various aspects of spatial ability. We evaluate the validity of a set of public-domain, algorithmically generated two-dimensional rotation items using a sample from the Synthetic Aperture Personality Assessment (SAPA) Project (*N* = 1,020,195). We examine the psychometric properties of the items and their relationship with various other cognitive abilities and personality traits. In addition, we identify the highest performing college majors and occupations on the 2D rotation items and on a set of 3D rotation items. Findings suggest strong unidimensionality for the 2D rotation items and the presence of lower-order factors which reflect differences across items in mental rotation demands. The highest scoring majors and occupations were similar—but not identical—across the 2D and 3D rotation measures and point to potentially meaningful differences across areas of expertise.

## 1. Introduction

Spatial abilities—which involve the generation, retention, retrieval, and transformation of well-structured visual images (Lohman 1996)—are needed for many everyday tasks such as map reading, navigation, and wayfinding (Hegarty et al. 2006; Pazzaglia and Moè 2013; Weisberg et al. 2014). Beyond these routine activities, spatial abilities are suggested to be involved in the more sublime, creative breakthroughs in STEM (i.e., science, technology, engineering, and mathematics; Kell and Lubinski 2013; Uttal and Cohen 2012). Nevertheless, spatial abilities are underrepresented in educational curricula, which remain heavily focused on developing reading, writing, and mathematical skills (Lohman 1988; Lubinski 2010). The overlooking of spatial abilities in the classroom is linked to the underachievement of spatially talented students, who may lack opportunities to develop their skills (Gohm et al. 1998; Lakin and Wai 2020). Despite their prominence in several popular frameworks of cognitive ability (Carroll 1993; Guilford 1967; Vernon 1950), spatial abilities are also underassessed in contexts such as academic achievement tests and in the identification of intellectually precocious youth (Shea et al. 2001; Webb et al. 2007). As a consequence, about half of the students scoring among the top 1% in spatial reasoning are believed to be unidentified in searches for high-aptitude scholars. This indicates that many spatially talented students do not develop their skills further, possibly resulting in a great loss of potential (Wai et al. 2009).

One barrier to assessing and developing spatial abilities is a relative lack of affordable measures. To address this concern, the central goal of the present work is to report on the development of a public-domain spatial ability measure—specifically, of the ability to mentally rotate two-dimensional (2D) figures. We evaluate the factor structure of a set of 2D rotation items and examine their relationship with various other cognitive abilities and personality traits. To evaluate the discriminant validity of the 2D rotation items, we examine how 2D and 3D rotation abilities vary differentially across areas of expertise by comparing their relationships to choice of college major and occupation. In addition, we examine the specific features of occupations most strongly related to 2D (and 3D) rotation skills using job characteristic ratings from the O*NET (National Center for O*NET Development 2022). These comparisons between 2D and the 3D rotation measures could be informative for two reasons: first, because the findings could provide support for the construct validity of the 2D rotation measure through a moderate to strong correlation with 3D rotation and similar (though not identical) associations with choice of major and occupation; second, because the complexity of this 2D rotation task distinguishes it from others, and unexpected findings could indicate that these items require a different strategy (or combination of strategies) to solve. The present work aims to report on the nomological network and psychometric properties of the 2D rotation task, but identifying and comparing the processes used to solve 2D and 3D rotation measures would be a valuable objective for future studies. We begin with a brief review of findings on the structure of spatial ability and its relationship to various individual differences and occupational contexts.

### 1.1. The Structure of Spatial Ability

Spatial ability can be parsed into related but distinct subcomponents, although a lack of consensus persists regarding the optimal factor structure (Buckley et al. 2018; Hegarty and Waller 2004). The absence of a coherent taxonomy of spatial abilities results in frequent muddling of labels given to abilities. Nonetheless, similarities emerge between the factors proposed across several popular frameworks of cognitive ability (Carroll 1993; Guilford 1967; Vernon 1950). Typically, these include variants on the ability to (a) solve problems by mentally rotating complex images (i.e., “spatial relations” or “mental rotation”); (b) manipulate visual patterns, such as those seen in paper-folding tasks (i.e., “spatial visualization” or “imagery quality”; Lohman 1979); (c) imagine an object from another perspective (i.e., “spatial orientation”; Hegarty and Waller 2005; Lohman 1996); and, more recently, (d) identify two-dimensional cross-sections of three-dimensional objects (Cohen and Hegarty 2012). Some frameworks include factors related to speed (e.g., a speeded rotation factor) or memory (e.g., visual memory; Schneider and McGrew 2012). Other lines of research suggest that spatial abilities can be further mapped along the dimensions of “static-dynamic” (the amount of movement of the stimulus; Contreras et al. 2003; Newcombe and Shipley 2015) and “intrinsic-extrinsic” (discerning the properties of specific objects vs. the interrelations among several objects; Uttal et al. 2013).

The present study focuses on mental rotation, a cognitive process through which one imagines how a 2D or 3D object would appear after it has been turned around a point by a certain angle (Shepard and Metzler 1971). Most studies linking spatial ability to STEM outcomes rely on measures of mental rotation (Buckley et al. 2018). Findings suggest that 3D rotation is most predictive of success in STEM as a broad domain, but success in certain subfields could depend more on related abilities, such as 2D rotation (e.g., which is important in chemistry; Wu and Shah 2004). Several researchers further differentiate between mental rotation abilities such as the ability to quickly rotate two-dimensional stimuli (“speeded rotation”), the ability to rotate three-dimensional stimuli, and the ability to imagine a stimulus from a different viewpoint (“perspective taking” or “spatial orientation”; Buckley et al. 2018; Hegarty and Waller 2004). The extent that relationships between these abilities and outcomes differ across fields—which could reflect the different importance of each skill across educational and occupational contexts—remains unclear. Clarifying which spatial abilities are most important in specific contexts within and beyond STEM will require the availability of valid assessment tools.

### 1.2. Relationships with Other Individual Differences

Considering spatial ability broadly, prior findings have been reported across multiple domains of psychological individual differences including cognitive ability, personality, and interests. Across all of these individual differences, the strongest relationships are found with mathematical abilities (Hegarty and Kozhevnikov 1999; van Garderen 2006) and other visual–perceptual abilities assessed by tasks such as matrix reasoning (Johnson and Bouchard 2005). The relationships between spatial ability and other cognitive abilities, such as verbal ability, tend to be weaker. In the domain of personality, spatial ability—like other cognitive abilities—is generally unrelated to any of the widely studied “broad-bandwidth” traits (i.e., the big five), with the exception of openness to experience/intellect, which tends to show a small positive relationship with spatial ability (von Stumm and Ackerman 2013). Some work also suggests a positive relationship between spatial ability and introversion (Lohman 1996), but this finding has not been widely replicated.

More findings support a relationship between spatial ability and occupational interests that reflects a preference for working with “things” rather than with “people” (Lubinski 2020; Prediger 1982). Spatially talented individuals often prefer “realistic” tasks (i.e., work requiring physical skill) and “investigative” tasks (i.e., research and knowledge building) and often dislike “social” tasks (i.e., work that involves working with or assisting others; Ackerman and Heggestad 1997; Lubinski 2020). These findings echo the claims that spatial ability influences learning and the development of expertise (Lubinski 2010; Wai et al. 2009), perhaps by predisposing individuals to develop these particular interests.

Much of the research on individual differences related to mental rotation, specifically, focuses on gender differences. Studies generally support a male advantage in mental rotation tasks—particularly of three-dimensional objects (Linn and Petersen 1985; Voyer et al. 1995)—but the origins of these differences are unknown. Prenatal androgen exposure, which is higher among males, is suggested as one explanation for these differences. The 2D:4D digit ratio, a biomarker of prenatal androgen exposure (Brown et al. 2002), often shows a relationship with mental rotation (Hampson et al. 2008). In addition to biological factors, some studies find support for the role of environmental influences such as gender roles and expectations in the development of spatial ability (Baenninger and Newcombe 1995; Voyer et al. 2000). Others suggest that differences in mental rotation can be explained by the use of different strategies, on average, by men and women (Linn and Petersen 1985; Voyer 2011). Efficient mental rotation is thought to be carried out analogously to physical rotation of an object; men tend to solve mental rotation problems this way spontaneously, whereas women appear more likely to rely on strategies involving “part-by-part” rotation for complex figures or to take a more analytical approach (Linn and Petersen 1985).

Considering 2D rotation ability specifically—sometimes referred to as “speeded rotation” and assessed under timed conditions (Lohman 1988)—a strong relationship is often found with other spatial abilities (Buckley et al. 2018; Burton and Fogarty 2003; Moreau 2013). Burton and Fogarty (2003) examined the structure of imagery and spatial abilities and found moderate to strong correlations for 2D rotation (the card rotation test) with tasks involving 3D rotation (cube comparison test, *r* = 0.58; spatial relations test, *r* = 0.77). These tests of mental rotation formed a “spatial relations” factor in Burton and Fogarty’s (2003) exploratory factor analysis, which correlated strongly (*r* = 0.77) with the “spatial visualization” factor (i.e., the paper-folding, paper form board, and surface development tasks). To date, very little prior work focused specifically on the relationship that 2D rotation shows with other individual differences, such as personality traits, relative to other spatial abilities. One potential explanation for the somewhat limited focus of prior work on 2D rotation could be that existing tasks are relatively simple. Two-dimensional rotation tasks tend to be less challenging than 3D rotation tasks, which could be one reason why they are often administered using a shorter time limit (i.e., in the case of “speeded rotation” tasks). It is not yet clear whether the existing set of 2D rotation tasks can be expanded to include more complex tasks developed for administration in untimed conditions—which is more typical when administering 3D rotation tasks.

### 1.3. Spatial Ability and Occupational Outcomes

Previous studies reveal the distinct importance of spatial ability for success in certain kinds of occupations (Hegarty and Waller 2005; Shea et al. 2001). Early studies on this topic examine the role of spatial ability in aviation and piloting, particularly among military personnel (Dror et al. 1993; Guilford and Lacey 1947; Humphreys and Lubinski 1996), and in careers that require high levels of mechanical reasoning (Smith 1964). In recent decades, the focus of research on spatial ability and occupational outcomes centered mainly on STEM-related tasks and work settings. Studies report moderate to strong correlations between psychometric tests of spatial ability and selection into and achievement in STEM careers, even while controlling for mathematical and verbal abilities (Kell et al. 2013; Shea et al. 2001; Uttal and Cohen 2012).

The importance of spatial ability is particularly evident among STEM careers such as geology (Hambrick et al. 2012), medicine and dentistry (Hegarty et al. 2009), and chemistry (Stieff 2007). These occupations routinely involve spatially demanding tasks such as mapping geological structures, constructing representations of mathematical problems, and performing surgical procedures (Hambrick et al. 2012; Hegarty and Waller 2005). At the height of STEM achievement, breakthroughs such as the discovery of the structure of the DNA molecule are also thought to result, at least partially, from high levels of spatial ability, which dispose individuals to more experiences of “spatial insight” (Uttal and Cohen 2012). Taken together, past findings portray spatial aptitude as the mainspring of achievement and innovation in STEM, only declining in relative importance as experts gain experience and rely increasingly on domain-specific knowledge (Uttal and Cohen 2012; Wai et al. 2009).

Some of the interest in understanding how spatial abilities relate to occupational outcomes arises from the growing need to recruit and prepare individuals to succeed in STEM-related careers (Xue and Larson 2015). To address these concerns, some researchers suggest allocating more focus toward understanding the spatial abilities demanded in highly specific contexts instead of assessing decontextualized spatial skills (e.g., mental rotation; Atit et al. 2020). A complementary approach could be to examine the relationships between commonly used measures of spatial ability and specific occupational features, or “job characteristics” along which occupations can vary. The job characteristics described in the O*NET content model (shown in Figure S20)—which include various aptitudes, interests, and interpersonal and physical skills—were cataloged as part of a job analysis of US occupations undertaken by the Bureau of Labor Statistics. Ratings for thousands of occupations on the importance of over 200 job characteristics describing skills of the typical worker (e.g., “analytical thinking”, “written expression”) and the working environment (e.g., “contact with others”, “deal with unpleasant or angry people”) are publicly available on the O*NET platform (Levine and Oswald 2012; Handel 2016). In alignment with the suggestions made by Atit et al. (2020), examining the relationship of these occupational features with various spatial ability tasks is expected to provide clarity regarding the factors that influence spatial aptitude in specific contexts.

### 1.4. The Present Study

This study has two central goals. The first is to validate a recently developed, public-domain measure of 2D rotation ability. This measure and the Item Response Theory (IRT) parameters for each item will be made available to researchers as part of the International Cognitive Ability Resource (ICAR) https://icar-project.com/ (accessed on 22 May 2023). Validation involves assessing the factor structure and unidimensionality of the 2D rotation items in addition to their relationship with various other individual differences. A second objective is to explore potential differences between 2D rotation ability and 3D rotation ability across occupational contexts. These findings offer preliminary evidence as to whether the 2D and 3D rotation items are solved via similar processes or whether further work is needed to understand how each task is typically solved.

## 2. Materials and Methods

### 2.1. Participants

Participants were a subset of the 1,558,035 individuals who responded to questions on the Synthetic Aperture Personality Assessment (SAPA) Project website https://www.sapa-project.org (accessed on 22 May 2023) between 7 February 2017 and 26 February 2023 in exchange for information about their personalities. Participants were not financially compensated for their involvement. The subset consisted of the 1,020,195 participants who self-reported having a high level of English fluency. The age of participants in this subsample (*M* = 26.8, *SD* = 12.8) ranged from 14 to 90 years. A total of 233 nation-states WERE represented, with 27.8% of participants residing in the United States. Tables with complete breakdowns for gender, ethnicity (for US participants only), and educational attainment of the sample are included in the supplementary material (Tables S1–S4). The dataset with all variables needed to run the major analyses reported in this manuscript are available on https://osf.io/afxdw/ (accessed on 22 May 2023).

### 2.2. Procedure

The SAPA Project uses a planned missingness design (Condon and Revelle 2015; Revelle et al. 2017), meaning that participants are administered a subset of items at random from a larger pool of thousands. All participants begin by reporting their demographic information and then responding to up to 250 items assessing individual differences across the domains of personality, cognitive ability, and interests. For the 2D rotation items in particular, participants in the present study were included if they responded to at least 1 of the items. A total of 454,714 of the participants responded to only one item, but note that many of these participants (though not all) were only administered a single item. Most participants in the sample (97%) were administered 3 or fewer of the 2D rotation items. After completing the survey, participants were able to view customized information about their personalities based on responses to the subset of items they answered from the SAPA Personality Inventory (Condon 2018). The data collection procedures used for the current study were reviewed by the Institutional Review Board at [masked for review]. For more details regarding the protocol, see Condon et al. (2017).

### 2.3. Measures

**Figure 1:**
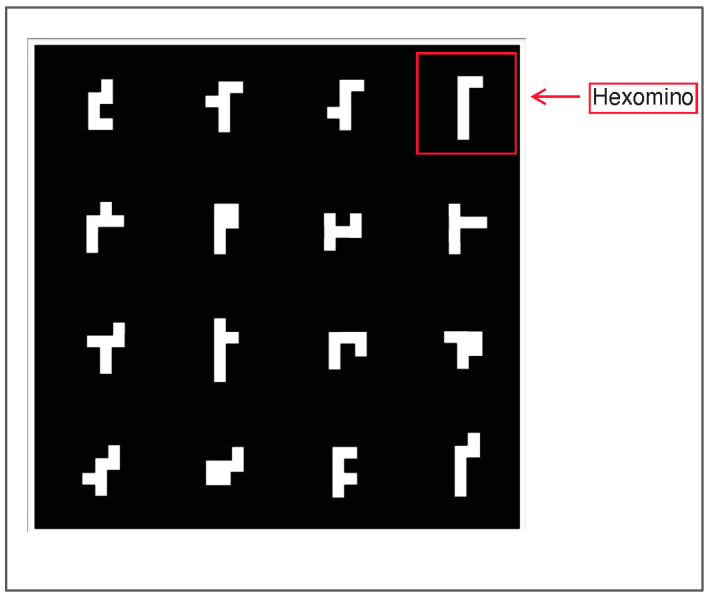
Example 4 × 4 array used as item stimuli.

**Figure 2:**
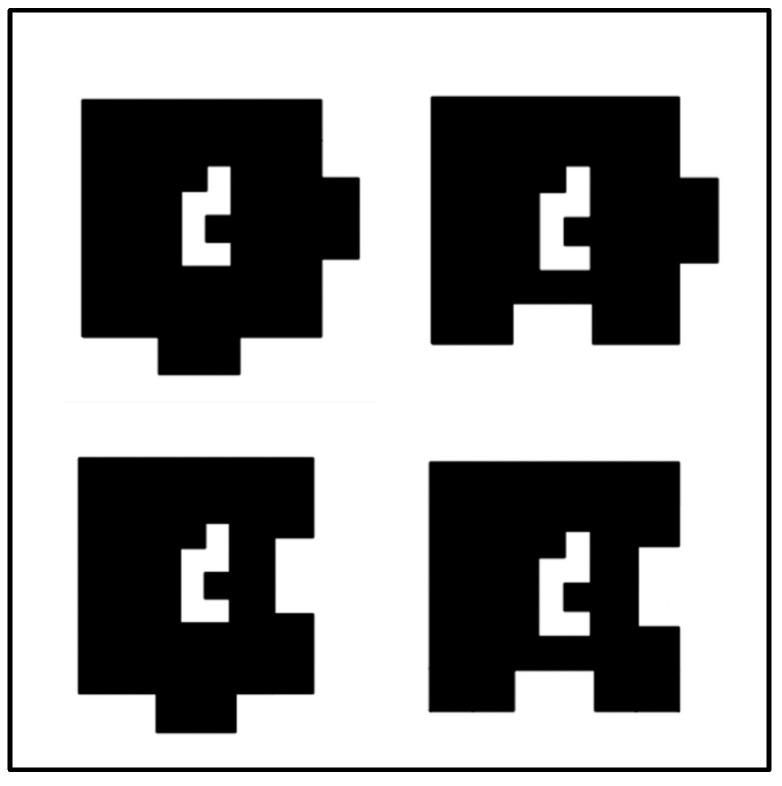
Four possible shapes for corner pieces.

**Figure 3:**
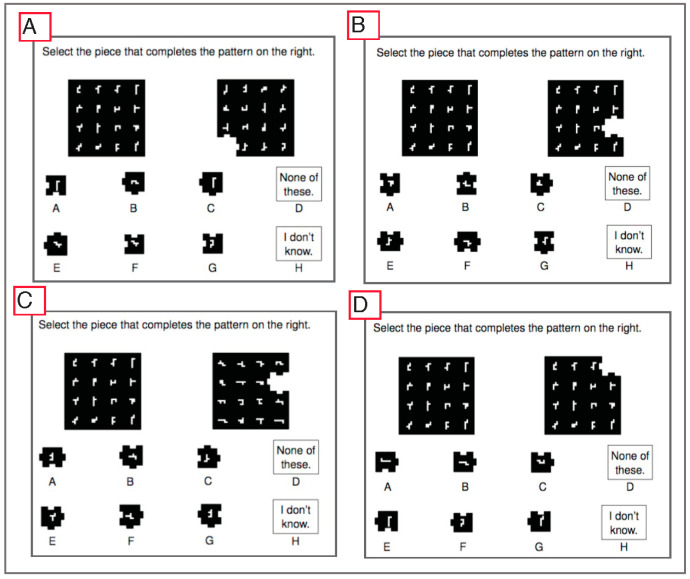
Examples for each of the four types of two-dimensional rotation items which (**A**) require rotation of both the reference object and answer option, (**B**) require rotation of the answer option only, (**C**) require rotation of the reference object only, or (**D**) do not require any rotation.

Two-dimensional rotation items: Participants were administered at least 1 item from a 70-item pool of 2D rotation items. These 70 items were chosen from a larger pool of 304 preliminary items based on the intention of covering a wide range of task difficulty (see the supplemental material for more information). Each of the items presented participants with a large square containing 16 small shapes in a 4 × 4 array (shown in Figure 1). To the right of this square, the same square was shown rotated 0–3 times by 90 degrees, with one of the corner pieces missing from the frame (the four possible shapes for corner pieces are shown in Figure 2). Participants were required to select the answer that completed the figure from a set of multiple-choice options. Six of the eight multiple-choice options showed a puzzle piece with 1 of the 16 small shapes, and the remaining two allowed participants to indicate that they did not know the answer or that the correct answer was not included among the answer options. Each item required either rotation of the large square, rotation of the answer option, rotation of both the large square and answer option, or rotation of neither (i.e., simple pattern matching). An example of each item type is displayed in Figure 3.

Several design features were considered during the development and initial testing of these items. Some decisions were based on the intention to minimize the extent to which performance on the items may be conflated with abilities other than two-dimensional rotation ability. For example, the items use only black-and-white coloring and a limited number of similarly sized, orthogonally oriented objects in order to minimize the influence of visual acuity and color–vision proficiency on performance. Of note, these design choices did not fully address all sources of conflation in that they did not overcome high levels of visual impairment and, perhaps more importantly, they had the disadvantage of causing the items to be relatively boring in comparison to more colorful and complex options.

Several other design decisions were based on the intention to develop a large and expandable pool of items using algorithmic rules. The reference object for each item (Figure 1) was derived by first identifying 16 visually distinct “hexominoes”, which are polygons made using 6 equally sized squares (also referred to as “polyominoes of order 6”) from the full set of 35 possible hexomino shapes. The reference object (shown in Figure 1) shows these 16 hexominoes arranged randomly into a 4 × 4 array. Only the single instance of ordering shown in Figure 1 has been used thus far, but a practically infinite number of permutations is possible based on these 16 objects (20 × 10^12^) and/or based on the potential use of alternate subsets of the 35 hexomino options. The target object to the right of the stimuli (see Figure 3 for examples) is a clockwise rotation of the stimuli (0, 90, 180, or 270 degrees) with 1 of the 16 hexominoes missing.

At the first stage of development, we began with the arrangement described above using square missing pieces—a 64-item set which was originally doubled (to 128 items) by also using targets that were inversions (i.e., mirror images) of the stimuli. The use of inversions was abandoned after early piloting, however, as it became clear that these items were invoking rotation along a 3rd dimension. Thus, the items were revised into their current form, using the puzzle-piece design for each of the missing pieces. In this version, there are several possible shapes for each missing piece, with the number of options depending on its location in the 4 × 4 array: corner pieces have 4 possible shapes (i.e., Figure 2), noncorner border pieces have 8 possible shapes, and internal pieces have 16 possible shapes. This implies that there are 144 uniquely shaped targets with one missing piece; in combination with the 4 possible rotations, this produces 576 possible stimuli:target pairings.

It should also be noted that the difficulty of correctly identifying the missing piece may be affected by features of the response option set. As described previously, all of the items in the current study use an 8-option response format, where 6 of the options are drawn from the set of 144 possible missing pieces. For all items, 1 choice is the correct answer, 1 choice is a distractor (also referred to as a competitor) with the correct hexomino piece of the incorrect shape (i.e., puzzle piece), and the remaining 4 are drawn randomly from the remaining pieces of the same shape type (e.g., corner, border, internal). To summarize, the design features for these items lead to 576 possible stimuli:target pairings when 1 of the 16 pieces (with 144 unique shapes) is missing from 1 of 4 possible rotations of the target. However, these 576 pairings can be used to generate many more possible items based on variations in the response-choice options. Similarly, the same materials can be permuted algorithmically to generate an inestimably large number of similar stimuli:target pairings.

**Figure 4:**
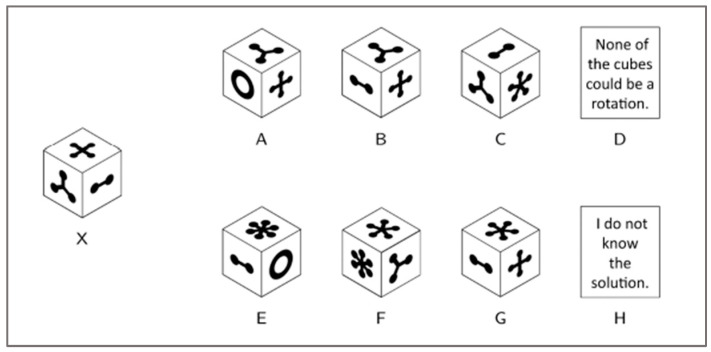
Example three-dimensional rotation item. Participants are asked to indicate which of the answers could be a possible rotation of the cube labeled “X”.

Cognitive ability: Several other cognitive abilities were assessed using items from the International Cognitive Ability Resource (ICAR; The International Cognitive Ability Resource Team 2014). These included 3D spatial rotation, matrix reasoning, letter and number series problem solving, verbal reasoning, figural analogies (Blum et al. 2016), propositional reasoning (Gühne et al. 2021), and compound remote associates problem solving (Mather et al., forthcoming). The multiple-choice response mode used was consistent across these item types. That is, they included six potentially correct answer options and two answer options that allowed participants to indicate that they did not know the answer or that the correct answer was not included among the available options. An example of one of the three-dimensional rotation items is shown in Figure 4, and example items for each of the other types can be found on the ICAR website (https://icar-project.com/, accessed on 22 May 2023).

Personality: Personality was assessed using the SAPA Personality Inventory (SPI; Condon 2018)—a hierarchical and empirically derived assessment tool for personality. The SPI has a total of 135 items that can be used to generate scores for the big 5 personality traits (14 items per trait) and for a narrower set of 27 unidimensional traits (5 items per trait) that are related to, but empirically distinct from, the big 5 traits. That is, these 27 traits describe personality at a narrower level but are not nested within the broader big five traits, which differentiates this model from others that include lower-level “aspects” or “facets” nested within each of the big five (Costa et al. 1991; DeYoung et al. 2007).

Occupation and university major: Participants were asked to report their university major (from a total of 146 options) and occupation (from a total of 1027 options). This self-reported information was used to compare the top-scoring majors and occupations on 2D and 3D rotation ability. In addition, we assessed the job *characteristics* most strongly associated with 2D and 3D rotation ability using ratings for each occupation—available on the O*NET (Handel 2016; Levine and Oswald 2012)—for 247 job characteristics. These job characteristics were organized within the O*NET content model into six broad categories (for a depiction, see Figure S20). Characteristics falling under the worker characteristics, worker requirements, experience requirements, and occupational requirements categories were included in the analyses. Descriptors in these categories characterized the typical worker (e.g., cognitive abilities, occupational interests, work values, work styles) and important activities (e.g., “helping others” or “working with computers”) for occupations.

### 2.4. Analyses

We evaluated the factor structure and unidimensionality of the 2D rotation items using the omega function from the “psych” package (Revelle 2022). In addition to providing estimates of internal consistency (i.e., omega total, alpha), the “omega” function uses exploratory factor analysis to estimate general factor saturation (omega general; Revelle and Zinbarg 2008). This is the proportion of variance that can be explained by the general factor, which will be used as an index of unidimensionality. To the extent that evidence supports the specification of lower-order factors, we provided estimates for the variance explained by each of the lower-order factors. Such evidence may also be used to identify items that could be dropped in order to improve the structure of the remaining item set and to identify the reasons for inconsistency among the items.

IRT scores were obtained based on the final set of 2D rotation ability items using the “mirt” package (Chalmers 2012). IRT scores were also generated for the other cognitive abilities in the ICAR framework and for the SPI traits, based on calibrations from previously published works (Mather et al., forthcoming; Blum et al. 2016; Blum and Holling 2018; Condon and Revelle 2014; Gühne et al. 2021). Sum scoring was used to score the big five personality traits, which were assessed using the same items as the SPI traits and represent a broader level of the trait hierarchy.

To evaluate the job characteristics most strongly related to 2D and 3D rotation ability, we applied the BISCUIT technique (aka, cross-validated best subsets regression; Elleman et al. 2021) using the “bestScales” function from the psych package (Revelle 2022). BISCUIT (Best Items Scales that are Cross validated, Unit weighted, Informative, and Transparent) is a correlation-based statistical learning technique that was developed for use in datasets that have high levels of missingness. This technique is also preferable because of its high transparency relative to similar statistical learning techniques. We specified in the “bestScales” function that k-fold (k = 10) cross-validation should be used instead of bootstrap aggregation. The analysis was run for both 2D and 3D rotation ability, resulting in a scale with the items (i.e., job characteristics) most strongly related to each type of spatial rotation. All analyses were conducted using R (R Core Team 2022).

## 3. Results

To ensure sufficient power, items were required to have a minimum of 500 pairwise administrations. The complete pool of 70 2D rotation items was administered on the SAPA Project website until this minimum was reached (*min* = 511; *M* = 597.48; *Mdn* = 597; *SD* = 26.73). Regarding total administrations, each item was administered at least 18,505 times (*M* = 19,069.64; *Mdn* = 19,011; *SD* = 291.52). The supplemental file contains figures and tables displaying the total number of total administrations per item (Figure S1) and pairwise administrations across items (Figure S2 and Table S5).

The factor structure of the items was examined using the omega function and by visually inspecting interitem correlation plots with one to six factors extracted (shown in Figure S3). These interitem correlation plots indicated clear multidimensionality among the items, and evaluation of the factor structure using the omega function provided further support for the presence of several lower-order factors. The omega hierarchical value (i.e., general factor saturation) suggested that the single-factor model demonstrated poor unidimensionality (*ω_h_* = 0.47) and that a four-factor solution was optimal. When accounting for the specific variance associated with these four lower-order factors, unidimensionality of the general factor improved (*ω_h_* = 0.66).

**Figure 5:**
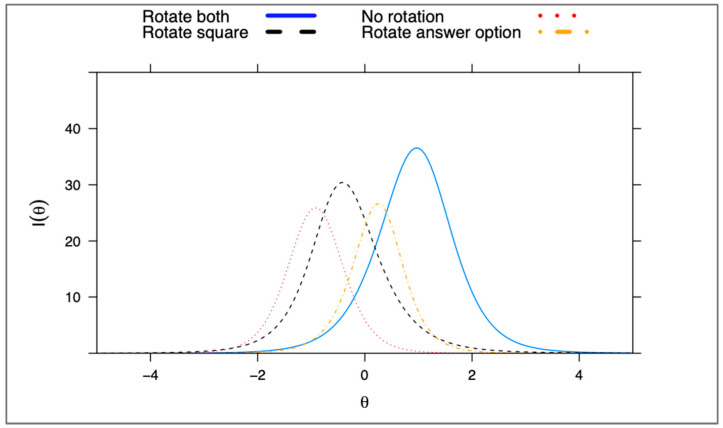
Test information for lower-order factors.

The four lower-order factors capture differences between the items in mental rotation demands. Most items had a strong primary loading onto one of these factors (shown in Table S6), which reflect the ability to (a) rotate both the stimuli and answer option; (b) rotate the answer option only; (c) rotate the stimuli only; or (d) identify the missing answer piece without any need for mental rotation. Examples of each of these item types are displayed in Figure 3, and a plot showing the relative difficulty of each item type (i.e., their test information curves) is shown in Figure 5 (and in Figure S15). This figure shows that the most informative and difficult items were those that required rotation of the stimuli and possible response choices. Items requiring no rotation were the easiest, and those requiring rotation of stimuli/response were moderately difficult. Because this task was developed primarily as a measure of 2D rotation ability, the 12 items that do not require any rotation were removed prior to generating IRT scores for general 2D rotation (leaving a total of 58 items). However, these items could still be useful for assessing other spatial skills, as they require some ability to detect visual patterns. After removing these items for the purpose developing a more coherent 2D rotation measure, unidimensionality improved (*ω_h_* = 0.77).

With sufficient evidence for unidimensionality of this subset of 58 items, IRT analyses based on a 2PL model were undertaken. After first running the analyses, one item appeared to be interfering with the overall test (shown in Figure S10), resulting in a test information curve with an unusual spike in reliability at middle levels of the latent trait (see Figure S9 and Table S7). This item had a high enough discrimination value to suggest that there could be an issue with the item. After removing this item and rerunning the analyses, the test information curve appeared as expected (see Figure S12), and this final set of 57 items was used to generate IRT scores for general 2D rotation ability. Items ranged in difficulty from −0.79 to 1.96 (*M* = 0.34; *SD* = 0.68) and in discrimination from 0.91 to 3.21 (*M* = 1.76; *SD* = 0.53). A table with difficulty and discrimination parameters for all 57 items can be viewed in the supplemental file (Table S8) and will be made publicly available on the ICAR website.

### 3.1. Relationship with Other Individual Differences

#### 3.1.1. Cognitive Abilities

Relationships between general 2D rotation ability and other cognitive abilities assessed using ICAR items are shown in Table 1 (and Figure S17). Correlations among all of the cognitive ability measures were similar (*r*’s = 0.17 to 0.32). Slightly stronger relationships were found for 2D rotation ability with matrix reasoning, letter–number series problem solving, and 3D rotation ability. Although the relationship for 2D rotation with 3D rotation was weaker than expected (*r* = 0.22), it was somewhat stronger than the relationships for 2D rotation with verbal ability (*r* = 0.21), compound remote associates problem solving (*r* = 0.17), and propositional reasoning (*r* = 0.19).

#### 3.1.2. Personality Traits

**Figure 6:**
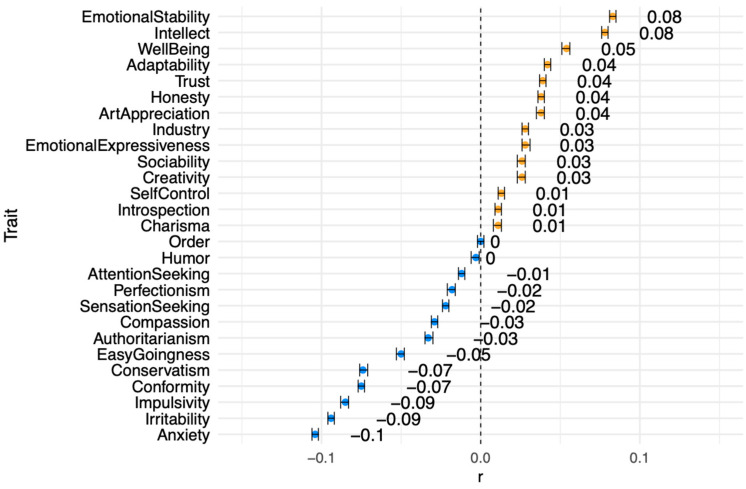
Correlations between 2D rotation and the SAPA Personality Inventory traits. Note: All correlations are significant at the *p* < .001 level except for order (*p* = .98) and humor (*p* = .002). All 95% confidence intervals ± |.005|.

As expected, 2D rotation ability showed weak relationships to the SPI traits (Figure 6 and Figure S18) and the big five traits (Table 2 and Figure S19). In line with previous findings, it was most strongly related to emotional stability and intellect in the positive direction (*r* = 0.08) and anxiety in the negative direction (*r* = −.10). There was no supporting evidence of the relationship proposed by Lohman (1988) between spatial ability and introversion (i.e., big five extraversion and sociability, charisma, and humor among the SPI traits in the present study).

### 3.2. Relationship to Job Characteristics and Occupational Choice

As anticipated, the O*NET job characteristics showing the strongest positive associations with 2D rotation ability (shown in Table 3 and Table S9) were related to STEM knowledge and skills. Most job characteristics showing the strongest negative associations with 2D rotation ability were related to interpersonal skills and frequent interaction with others. Other characteristics that showed negative associations with 2D rotation ability were related to physical labor.

The relationship between mental rotation ability and STEM-related tasks and activities was also supported by the associations between occupations and performance with the 2D rotation items. The occupations with the highest correlations (shown in Table 4 and Table S10) were almost exclusively STEM-related. There was also strong overlap between the occupations that scored highest on 2D rotation ability and the occupations that scored the highest on 3D rotation ability (shown in Table 5 and Table S11), which were also predominantly STEM-related. Still, there was some evidence for discriminant validity in the 2D and 3D rotation measures. Among the top 20 occupations listed in Table 4, electrical installers and tactical operations leaders (in the military) have notably higher percentile ranks in 2D rotation; engineers, by contrast, generally have higher percentile ranks on 3D rotation. Additional tables displaying the occupations with the greatest discrepancy between 2D and 3D rotation performance can be viewed in the supplemental file (Tables S12 and S13).

Because college majors are often chosen based on a planned career path (in addition to many other possible factors), we also examine the college majors that score the highest on 2D and 3D rotation abilities. These are shown in Table 6 and Table 7 below, and in the supplemental file (Tables S14–S17). The highest scoring college majors were very similar to the highest scoring occupations. However, it was somewhat more common for non-STEM-related fields (e.g., religion, music, philosophy) to be represented among the highest scoring majors than among the highest scoring occupations. These comparisons across majors on 2D and 3D rotation ability do not provide the same degree of support for discriminant validity demonstrated by the comparisons across occupations. However, taken together with the comparisons across occupations, these findings support the possibility that spatial ability and the development of expertise in various STEM fields are mutually reinforcing. This could explain why those in STEM occupations have a stronger advantage in spatial tasks than those currently majoring in STEM fields, who have not had as much time to develop spatially relevant skills.

## 4. Discussion

The present study evaluated the validity of a large set of algorithmically generated 2D rotation items. Findings indicate that the items demonstrate strong psychometric properties and provide evidence for strong (albeit preliminary) construct validity. We review these findings below and discuss possible explanations for several unexpected results of the present work. In addition, we identify some limitations of this study and future directions for the development and use of the 2D rotation items, and we review how researchers can make use of the IRT parameters.

### 4.1. Psychometric Properties

Regarding the structure of the items, analyses indicated the presence of four clear lower-order factors among the complete set of 70 items, reflecting variations in the type of mental rotation required (i.e., whether the items require rotation of the stimuli, answer option, both of these, or neither of these). After excluding the subset of 12 items that do not require any rotation of the stimuli or answer option and one low-quality item, the remaining 57 items demonstrate strong unidimensionality, as indicated by the high omega hierarchical value. In addition, IRT analyses revealed that most of the items are best at distinguishing among participants in the average range of 2D rotation ability. However, there was some variability in item difficulty for these items, suggesting that they are also appropriate for samples of higher or lower mental rotation ability. Item difficulty parameters for each item are available in Table S8.

### 4.2. Relationships with Other Cognitive Abilities

As expected, 2D rotation ability showed positive relationships with the other cognitive abilities assessed. The general pattern of correlations shown for 2D rotation ability with these other lower-order cognitive abilities echoed previous findings—stronger relationships emerged with the more visual item types (e.g., 3D rotation ability and matrix reasoning) than the more verbal item types (e.g., compound remote associates problem solving, verbal reasoning). However, the relationship between the 2D and 3D rotation measures was weaker than expected considering the high degree of similarity in the constructs they assess. One explanation could be that participants differ in the strategies they use to solve the 2D rotation items—some might rely on visually rotating the stimuli whereas others might take a more analytical approach. This distinction was highlighted elsewhere, as in the context of 3D rotation (Condon and Revelle 2014; Kail et al. 1979). It could also be the case that this particular 2D rotation task is solved using a more analytical strategy than is used for existing 2D rotation tasks. This is plausible considering that many of the items involve multiple steps (i.e., two objects to rotate) and all of the items require participants to detect and remain aware of the often subtle differences between shapes. Though evaluation of the processes used to solve the 2D rotation items was beyond the scope of the present work, this should be prioritized in future studies.

### 4.3. Relationships with Personality Traits

Reflecting previous findings, relationships for 2D rotation ability with personality traits were relatively weak. The present study examined relationships between 2D rotation and the big 5 traits as well as 27 narrower traits from the SAPA Personality Inventory. The strongest correlations emerged for 2D rotation with big five neuroticism and SPI anxiety. This aligns with the previous studies focusing on a variety of cognitive abilities which suggest that negative emotionality impairs focus and problem-solving ability (Eysenck et al. 2005; Eysenck 2013). Findings also demonstrated the utility of assessing personality at narrower levels than the broad big-five model allows. In the positive direction, 2D rotation was most strongly correlated with SPI emotional stability and SPI intellect. Big-five openness and other, narrower SPI traits related to openness (e.g., art appreciation, introspection) showed weaker correlations with 2D rotation.

### 4.4. Relationships Shown with Job Characteristics and Occupational Choice

Support was found for the relationship between mental rotation ability and STEM-related skills and occupational choices. Several O*NET job characteristics that are relevant to a wide range of occupations (e.g., analytical thinking, complex problem solving) were represented among the 20 job characteristics most positively associated with 2D rotation. However, most of the job characteristics that were most strongly related to 2D rotation ability represent skills and areas of knowledge that fall distinctly within the broad STEM domain (e.g., engineering and technology, physics knowledge, technology design, mathematics skills). It was notable that while several of the job characteristics most negatively associated with 2D rotation ability were related to physical labor (e.g., spend time making repetitive motions), most were related to interpersonal interactions (e.g., contact with others, assisting and caring for others, relationships, deal with unpleasant or angry people).

However, it is not necessarily the case that those with strong spatial skills struggle with these more interpersonal tasks, as many studies—including the present work—report weak relationships between mental rotation and interpersonally relevant traits (e.g., big-five extraversion and agreeableness; SPI sociability, charisma, and compassion). It could be that those with high mental rotation ability self-select into occupations that support the use of this skill, which might involve more solitary work and draw less on interpersonal skills. In Prediger’s (1982) model of vocational interests—which proposes two dimensions of “data/ideas” and “people/things”—these results reflect a stronger preference for thing-related tasks rather than the people-related tasks. This finding also appears to be captured by Ackerman and Heggestad’s (1997) science/math “trait complex”. This trait complex reflects the covariation of math/visual perception abilities and investigative vocational interests, but, unlike other trait complexes, it does not include personality traits (e.g., the social trait complex includes enterprising/social interests as well as extraversion and social potency).

The relevance of 2D rotation ability to STEM is also supported by findings on the highest scoring occupations and college majors. Regarding occupations, those scoring the highest on 2D and 3D rotation ability were very similar, with most falling clearly within the domain of STEM. However, some differences emerged in the top-scoring occupations between these item types, which may be an indication of the discriminant validity of these measures. STEM fields were also represented among the college majors scoring the highest on 2D and 3D rotation abilities but not as strongly as with the top-scoring occupations. Several non-STEM-related fields (e.g., classical languages, religion) were included among the top-scoring majors. This likely reflects the tendency for frequently used skills, such as those demanded by one’s occupation, to strengthen over time relative to those of others who do not use these skills as frequently.

### 4.5. Limitations and Future Directions

Given the psychometric properties of the items and evidence for their validity, researchers may be interested in using this instrument in their own work. We identify several limitations of the present work and possible future directions that might be of interest to other researchers. Note that, depending on the use case, IRT-based scoring procedures may be particularly useful (i.e., in cases where there is only space to administer a few items or when seeking to evaluate across a wide range of 2D ability). For these cases, the IRT parameters identified in the Supplemental Materials (Table S8) can also be obtained from the ICAR website.

One limitation of the present work is that we could not adjudicate whether the strategies used to solve the 2D rotation items mirror those used to solve the 3D rotation items. This is relevant to several findings from this study, and seems particularly important to the potential development of 2D skills. Future work evaluating the processes used to solve each item type is needed, and this may require more qualitative data collection methods such as cognitive interviewing. In addition, researchers might consider more closely examining the item types in each of the lower-order 2D rotation factors found in the present work. It is unclear whether these item types—which differ in how much mental rotation is demanded—vary only in difficulty or if there are potentially meaningful differences in the strategies that are most useful for solving them. Additionally, although the dataset used in the present work is highly representative of the general population, participants found and decided to take the online survey (presumably out of interest) rather than being actively recruited by the researchers. It is unclear whether these participants differ in any systematic ways from a representative sample recruited via random sampling processes (e.g., for reasons relating to internet access or interest in psychology). While truly random sampling techniques are somewhat uncommon, further work could evaluate these items using other data sources, such as community or university samples from specific regions.

## 5. Conclusions

The present study provided support for the validity and unidimensionality of a set of 57 2D rotation items. In addition, findings suggest that the preliminary set of 69 2D rotation items—which include an additional 12 items that only require pattern matching—can be divided into subsets and used to evaluate four more specific 2D rotation skills. IRT analyses suggested that the items vary moderately in difficulty, which makes them suitable for samples across a broad range of anticipated mental rotation abilities. Although further work is needed to understand how the 2D rotation items are usually solved (i.e., through mental rotation or other, more analytical strategies), there also appears to be some noteworthy differences in the occupational choice of top 2D rotation performers and top 3D rotation performers that are supported by previous findings. This relatively challenging 2D rotation task could be used for identifying the occupations in which 2D rotation is especially important, which is not well understood relative to findings on the role of 3D rotation in various occupations.

## Figures and Tables

**Table 1:** Correlations between 2D rotation and other cognitive abilities.

Variable	*M*	*SD*	1	2	3	4	5	6	7	8
1. Two-dimensional rotation	50	10	-							
2. Figural analogies	49.70	5.50	0.20	-						
3. Propositional reasoning	49.39	6.21	0.19	0.24	-					
4. Letter–number series	47.76	6.62	0.24	0.26	0.27	-				
5. Three-dimensional rotation	49.51	5.85	0.22	0.22	0.20	0.23	-			
6. Matrix reasoning	48.79	5.34	0.23	0.25	0.22	0.32	0.22	-		
7. Verbal reasoning	47.91	5.88	0.21	0.20	0.26	0.32	0.21	0.25	-	
8. Compound remote associates	50	5.98	0.17	0.18	0.23	0.24	0.19	0.19	0.25	-

Note: All correlations are significant at the *p* < .001 level. All 95% confidence intervals ± |.003|.

**Table 2:** Correlations between 2D rotation and the big five traits.

Variable	*M*	*SD*	1	2	3	4	5	6
1. Two-dimensional rotation	50	10	-					
2. Agreeableness	4.22	0.88	0.04	-				
3. Conscientiousness	3.95	0.97	0.02	0.21	-			
4. Extraversion	3.31	1.11	0.01	0.14	0.08	-		
5. Neuroticism	4.08	1.08	−0.10	−0.09	−0.20	−0.16	-	
6. Openness	4.58	0.81	0.05	0.09	0.16	0.10	−0.05	-

Note: All correlations are significant at the *p* < .001 level. All 95% confidence intervals ± |.005|.

**Table 3:** Top 40 job characteristics associated with 2D rotation.

	Frequency	Mean Correlation	Standard Deviation
**20 strongest positive associations**			
Engineering and technology	10	0.52	0.02
Investigative	10	0.51	0.01
Physics knowledge	10	0.48	0.02
Technology design	10	0.48	0.02
Science	10	0.48	0.01
Mathematics skills	10	0.48	0.01
Mathematical reasoning	10	0.47	0.01
Mathematics knowledge	10	0.47	0.01
Design	10	0.46	0.02
Analytical thinking	10	0.46	0.01
Systems analysis	10	0.45	0.01
Estimating the quantifiable characteristics of products, events, or information	10	0.45	0.01
Information ordering	10	0.45	0.02
Programming	10	0.44	0.01
Category flexibility	10	0.44	0.02
Number facility	10	0.43	0.01
Operations analysis	10	0.42	0.01
Complex problem solving	10	0.42	0.01
Analyzing data or information	10	0.42	0.01
Visualization	10	0.39	0.02
**20 strongest negative associations**			
Therapy and counseling	10	−0.20	0.01
Enterprising	9	−0.20	0.02
Stress tolerance	10	−0.21	0.01
Physical proximity	10	−0.21	0.01
Social perceptiveness	10	−0.22	0.01
Spend time making repetitive motions	10	−0.23	0.01
Deal With physically aggressive people	10	−0.26	0.01
Clerical	10	−0.26	0.01
Assisting and caring for others	10	−0.27	0.01
Service orientation	10	−0.29	0.01
Contact with others	10	−0.31	0.02
Deal with external customers	10	−0.32	0.02
Social	10	−0.32	0.01
Self-control	10	−0.33	0.01
Concern for others	10	−0.34	0.01
Relationships	10	−0.35	0.01
Social orientation	10	−0.35	0.01
Performing for or working directly with the public	10	−0.37	0.01
Customer and personal service	10	−0.38	0.02
Deal with unpleasant or angry people	10	−0.40	0.01

**Table 4:** Twenty highest scoring occupations on 2D rotation (in descending order of 2D rotation percentile).

Occupation	N	R2D	R3D	Difference
Chemical engineer	606	0.91	0.93	−0.02
Chemist	595	0.91	0.92	−0.01
Aerospace engineer	567	0.90	0.96	−0.06
Biologists	720	0.90	0.91	−0.01
Computer systems engineer/architect	1791	0.89	0.95	−0.06
Mechanical engineer	1457	0.89	0.91	−0.02
Network and computer systems administrator	1099	0.86	0.88	−0.02
Web developer	1349	0.84	0.92	−0.08
Computer software engineer	6373	0.83	0.93	−0.1
Automotive engineer	539	0.83	0.84	−0.01
Architect	2214	0.82	0.81	0.01
Electrical engineer	1329	0.82	0.86	−0.04
Engineering manager	851	0.82	0.93	−0.11
Business intelligence analyst	1640	0.81	0.88	−0.07
Electronics engineer	821	0.81	0.86	−0.05
Veterinarian	560	0.81	0.82	−0.01
Electrical and electronics installer and/or repairer	605	0.80	0.67	0.13
Computer and information systems manager	1271	0.80	0.90	−0.1
Other—military officer special and tactical operations leader/manager	839	0.80	0.72	0.08
Computer programmer	4666	0.78	0.91	−0.13

**Table 5:** Twenty highest scoring occupations on 3D rotation (in descending order of 3D rotation percentile).

Occupation	N	R2D	R3D	Difference
Aerospace engineer	567	0.90	0.96	−0.06
Computer systems engineer/architect	1791	0.89	0.95	−0.06
Computer software engineer	6373	0.83	0.93	−0.1
Chemical engineer	606	0.91	0.93	−0.02
Engineering manager	851	0.82	0.93	−0.11
Web developer	1349	0.84	0.92	−0.08
Chemist	595	0.91	0.92	−0.01
Computer programmer	4666	0.78	0.91	−0.13
Mechanical engineer	1457	0.89	0.91	−0.02
Biologist	720	0.90	0.91	−0.01
Computer and information systems manager	1271	0.80	0.90	−0.1
Business intelligence analyst	1640	0.81	0.88	−0.07
Computer security specialist	786	0.73	0.88	−0.15
Network and computer systems administrator	1099	0.86	0.88	−0.02
Electrical engineer	1329	0.82	0.86	−0.04
Electronics engineer	821	0.81	0.86	−0.05
Information technology project manager	2273	0.77	0.85	−0.08
Automotive engineer	539	0.83	0.84	−0.01
Management analyst	1807	0.73	0.82	−0.09
Veterinarian	560	0.81	0.82	−0.01

**Table 6:** Twenty highest scoring majors on 2D rotation (in descending order of 2D rotation percentile).

Major	N	R2D	R3D	Difference
Neuroscience	1269	1	1	0.00
Applied mathematics	1579	0.99	0.98	0.01
Physics	5664	0.99	0.99	0.00
Materials science and engineering	1470	0.98	0.96	0.02
Biomedical engineering	2413	0.97	0.97	0.00
Manufacturing and design engineering	1733	0.96	0.73	0.23
Mathematics	6953	0.95	0.94	0.01
Chemical and biological engineering	5797	0.94	0.92	0.02
Mechanical engineering	14,670	0.93	0.88	0.05
Aerospace engineering	2682	0.92	0.89	0.03
Anthropology	2438	0.91	0.93	−0.02
Geological sciences	2512	0.90	0.85	0.05
Industrial engineering	3834	0.88	0.90	−0.02
Chemistry	8269	0.88	0.86	0.02
Actuarial sciences	941	0.86	0.88	−0.02
Biology	19,049	0.86	0.86	0.00
Other mathematics major	896	0.85	0.79	0.06
Landscape design	587	0.84	0.64	0.20
Religion	987	0.83	0.84	−0.01
Electrical engineering	16,634	0.82	0.77	0.05

**Table 7:** Twenty highest scoring majors on 3D rotation (in descending order or 3D rotation percentile).

Major	N	R2D	R3D	Difference
Neuroscience	1269	1	1	0.00
Physics	5664	0.99	0.99	0.00
Applied mathematics	1579	0.99	0.98	0.01
Biomedical engineering	2413	0.97	0.97	0.00
Materials science and engineering	1470	0.98	0.96	0.02
Philosophy	2818	0.81	0.95	−0.14
Mathematics	6953	0.95	0.94	0.01
Anthropology	2438	0.91	0.93	−0.02
Chemical and biological engineering	5797	0.94	0.92	0.02
Music	3989	0.79	0.91	−0.12
Industrial engineering	3834	0.88	0.90	−0.02
Aerospace engineering	2682	0.92	0.89	0.03
Mechanical engineering	14,670	0.93	0.88	0.05
Actuarial sciences	941	0.86	0.88	−0.02
Statistics	2174	0.77	0.87	−0.10
Biology	19,049	0.86	0.86	0.00
Chemistry	8269	0.88	0.86	0.02
Geological sciences	2512	0.90	0.85	0.05
Religion	987	0.83	0.84	−0.01
Music education	712	0.73	0.82	−0.09

## Data Availability

Data related to the primary variables evaluated in this work have been made publicly available on the project OSF page at https://osf.io/afxdw/ (accessed on 22 May 2023). Additional data used for validation against external variables are part of a larger data collection effort (the SAPA Project) for which data are made publicly available at regular intervals at https://dataverse.harvard.edu/dataverse/SAPA-Project (accessed on 22 May 2023).

## Supplementary Materials

Supporting information for this work can be downloaded at https://www.mdpi.com/article/10.3390/jintelligence11100191/s1. Figure S1: Total number of administrations per item; Table S1: Educational attainment; Figure S2: pairwise administrations across item pairs; Table S2: Ethnicity (U.S. participants only); Figure S3: inter−item correlations for factor solutions with 1−6 factors; Table S3: Employment status; Figure S4: Example item for factor 1 (“rotate both”); Table S4: English fluency; Figure S5: Example item for factor 2 (“rotate answer”); Table S5: Pairwise admins for subset of 70 items; Figure S6: Example item for factor 3 (“rotate stimuli”); Table S6: Factor loadings; Figure S7: Example item for factor 4 (“rotate none”); Table S7: Item descriptives and IRT parameters, in descending order of item discrimination (potentially problematic items highlighted); Figure S8: Item probability functions; Table S8: Item descriptives and IRT parameters, in descending order of item discrimination; Figure S9: Test Information and Standard Errors; Table S9: 40 Job Characteristics Most Strongly associated with 2D Rotation; Figure S10: One item with an unusually high discrimination value; Table S10: Highest scoring occupations on 2D rotation (in descending order of 2D rotation percentile); Figure S11: Item Probability Functions; Table S11: Highest scoring occupations on 3D rotation (in descending order of 3D rotation percentile); Figure S12: Test Information and Standard Errors; Table S12: Occupations with the greatest difference in performance (in favor of 2D rotation); Figure S13: One other item (item 16144) had a fairly high difficulty level and a similar but less challenging distractor option; Table S13: Occupations with the greatest difference in performance (in favor of 3D rotation); Figure S14: Item Probability Functions; Table S14: Highest scoring majors on 2D rotation (in descending order of 2D rotation percentile); Figure S15: Test Information For Lower−order Factors; Table S15: Highest scoring majors on 3D rotation (in descending order of 3D rotation percentile); Figure S16: Correlations between lower−order abilities; Table S16: Majors with the greatest discrepancy in performance (in favor of 2D rotation); Figure S17: Correlations with other cognitive abilities; Figure S18: Correlation of 2D rotation with the 27 SPI traits; Table S17: Majors with the greatest discrepancy in performance (in favor of 3D rotation); Figure S19: Correlations with the big five traits; Figure S20: O*NET Content model.
