# Supplementary material for: Development of a Public-Domain Measure of Two-Dimensional Rotation Ability and Preliminary Evidence for Discriminant Validity among Occupations
Source: J Intell. 2023 Oct 3;11(10):191. doi: 10.3390/jintelligence11100191 (PMC10607440; doi:10.3390/jintelligence11100191)
Supplement: Supplementary file 1 [file jintelligence-11-00191-s001.zip › jintelligence-2438545-supplementary.pdf]

Two-Dimensional Rotation Ability Scale Development

[Masked for review], [Masked for review]

Contents

Loading packages and data **2**

Participants **2**

Identifying 2D rotation items with enough pairwise administrations **4**

Factor structure and dimensionality **7**

IRT analyses of the items **13**
- Item parameters for general factor . . . 13
- Lower-order factors . . . 20

Correlations between 2D rotation and other individual differences **23**
- Cogitive abilities . . . 23
- Temperament . . . 25
- O*NET job characteristics . . . 26
- Occupations and Majors . . . 28

Loading packages and data

The dataset ("ICARdata") contains IRT scale scores for the 27 traits of the SAPA Personality Inventory (SPI) and cognitive ability scale scores based on the International Cognitive Ability Resource (ICAR), which includes measures of 3D rotation, verbal reasoning, associative ability, propositional reasoning, matrix reasoning, and letter-number series problem-solving. In addition, sum scores for the Big Five personality traits were generated using the SPI items.

```
library(psych)
library(tidyverse)
library(here)
library(rio)
library(mirt)
library(latticeExtra)
library(knitr)
library(kableExtra)
library(flextable)

options(scipen = 999)

# Loading data with IRT scores for R2D
#load("~/Desktop/Projects/2D_Rotation/R2Ddata07feb2017thru26feb2023.rdata")
load("~/Desktop/Projects/2D_Rotation/ICARdata_06March.rdata")
```

Participants

First, participants are excluded from analyses if they are under the age of 14 or if they did not report speaking English at least "Well" (i.e., they selected "Not Well" or "Not at all, need translation").

```
ICARdata <- ICARdata %>%
  # Filter for participants who are at least 14
  filter(age>13) %>%
  # Filter for english fluency
  filter(english=="Well" | english=="VeryWellFluentNative")


totalN <- nrow(ICARdata)
```

This initial sample included a total of 1,558,035 participants. Below, the subset of participants who responded to at least 1 2D rotation item is identified.

```
ICARdata <- ICARdata %>%
  mutate(R2Dcount = rowSums(!is.na(.[86:389]))) %>% # Count the number of non NAs across the R2D items
  filter(R2Dcount > 0) # At least 1 non NA response
```

The final sample included a total of 1,020,195 participants (68.68 % female) who responded to items on the SAPA project website (www.sapa-project.org) in exchange for information about their personalities. The sample ranged in age from 14 to 90 years, with a mean of 26.8 ($sd = 12.8$). A total of 233 nation states were included (27.76% U.S.). Information on participant employment status, educational attainment, ethnicity (for U.S. participants only), and English fluency is shown in the tables below. Note that responses to these items were not required so the total number of participants included in these tables is less than the complete sample used for these analyses.

Table S1: Educational attainment

Educational Attainment	Participants	Percentage
Less than 12 years	93,923	10.4%
High school graduate	131,520	14.5%
Currently in college/university	246,058	27.2%
Some college/university, but did not graduate	54,141	6%
Associate degree	26,848	3%
College/university degree	177,844	19.6%
Currently in graduate or professional school	37,621	4.2%
Graduate or professional school degree	138,174	15.2%

Table S2: Ethnicity (U.S. participants only)

Ethnicity/Race	Participants	Percentage
White	138,186	73%
Two or more ethnicities	10,526	5.6%
African American	10,149	5.4%
Mexican American	9,415	5%
Other Hispanic	5,307	2.8%
Chinese	2,272	1.2%
Other Asian	1,914	1%
Puerto Rican	1,779	0.9%
Indian	1,760	0.9%
Filipino	1,666	0.9%
Native American	1,498	0.8%
Other	1,475	0.8%
Korean	1,052	0.6%
None of these	725	0.4%
Cuban American	567	0.3%
Japanese	500	0.3%
Other Pacific Islander	241	0.1%
Native Hawaiian	190	0.1%
Alaskan Native	129	0.1%

Table S3: Employment status

Job Status	Participants	Percentage
Currently a student	352,149	41.7%
Employed	333,146	39.4%
Not employed	72,376	8.6%
Not employed, seeking work	46,411	5.5%
Retired	25,204	3%
Homemaker	15,732	1.9%

Table S4: English fluency

English fluency	Participants	Percentage
Very well/fluent	591,868	58%
Well	428,327	42%
Need Translation	0	0%
Not Well	0	0%

Descriptive statistics for all 2D rotation items

```
# Subsetting the data for just the 2D rotation items
Rot2D <- subset(ICARdata, select = c(q_16001:q_16304))

# number of pairwise admins for all 304 item pairs
R2D_pairwise_counts <- pairwiseCount(Rot2D, diagonal=FALSE)
pwise_counts_table_unfiltered <- psych::describe(R2D_pairwise_counts)

R2D_pairwise_counts_summary <- as.vector(R2D_pairwise_counts
                               [lower.tri(R2D_pairwise_counts)])

psych::describe(R2D_pairwise_counts_summary)
```

```
##    vars     n  mean     sd median trimmed  mad min max range skew kurtosis   se
## X1    1 46056 36.69 132.09      6    5.79 2.97   0 691   691 4.03    14.27 0.62
```

Identifying 2D rotation items with enough pairwise administrations

There are a total of 304 algorithmically generated 2D rotation items. This large pool of initial items was generated to ensure that there would be a final subset of high-quality items that cover a broad range of difficulty (i.e., enough items that are relatively easy or difficult, which are needed to distinguish between participants at higher levels of 2D rotation ability and at lower levels of 2D rotation ability).

Once developed, all 304 items were administered a relatively small number of times ($m = 1226.19$); $Mdn =$ 1224; minimum = 1138) on the SAPA Project website in order to identify mean estimates of their difficulty based on the proportion of correct responses. In this way, a subset of the initial 304 items was identified for further testing and validation. The items reported on in the current study (N = 70) were then subsequently administered at much higher rates until they reached a minimum of 500 pairwise administrations with one another (i.e., among the smaller 70-item set, not the complete pool of 304 items). The histogram below shows the number of administrations for all 304 items, with the 70 items that were selected for further development at the high end of the range (>20,000 administrations) and the remainder at the low end.

```
# column sums to get total number of total administrations
admins <- as.data.frame(colSums(!is.na(Rot2D))) %>%
          dplyr::rename(admins = 1) %>%
          dplyr::arrange(desc(admins))
```

Figure S1. Total number of administrations per item

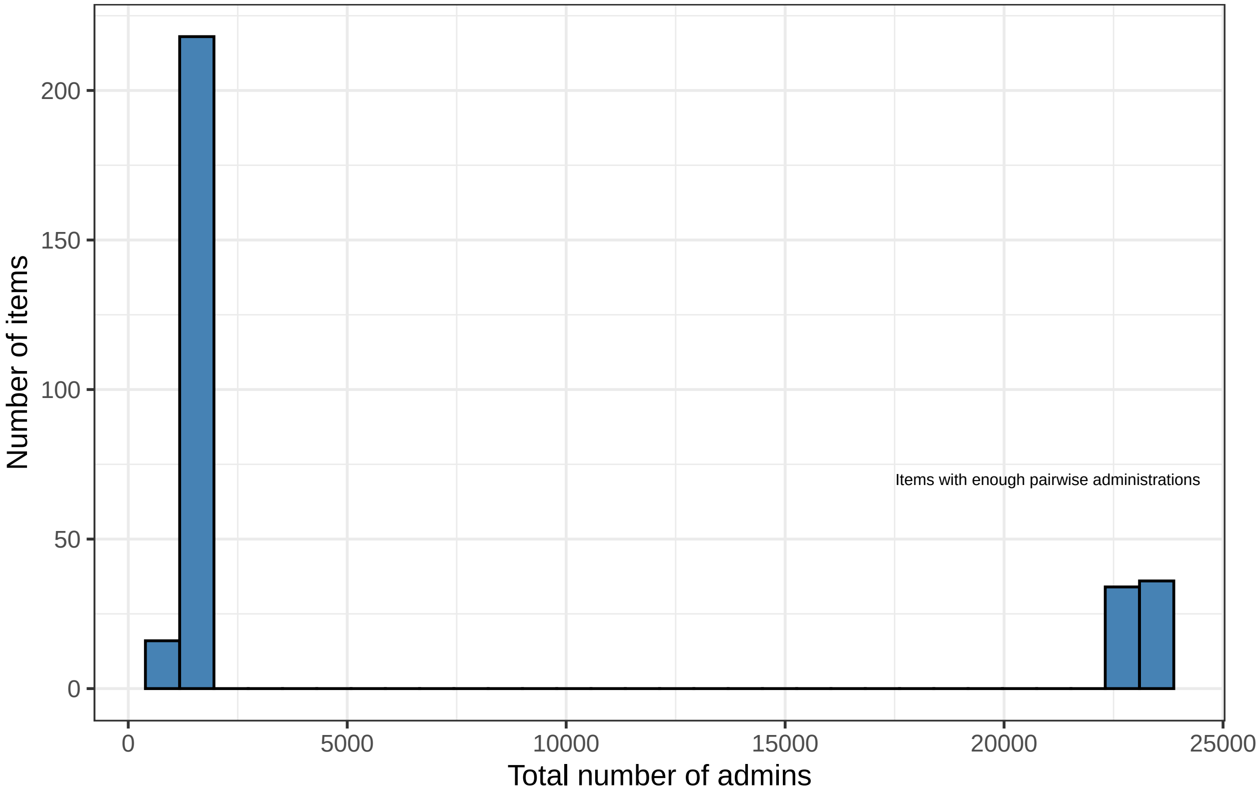

Most items (N = 234 out of 304) have not been administered frequently enough with each of the other items. We remove these items below and then re-evaluate the number of minimum pairwise administrations per item using the remaining 70 items. To be clear, the pairwise counts shown in table S5 are limited to these 70 items as a set.

```
# filtering dataframe of items
admin_rows <- admins %>% row.names()
R2D_item_nums <- admin_rows[1:70] # items with enough overall administrations
# Sub-setting the data to get only the items with enough admins
Rot2D_subset <- subset(Rot2D, select = c(R2D_item_nums))
# Getting pairwise administrations for these items
```

```
R2D_pairwise_counts_filter <- pairwiseCount(Rot2D_subset, diagonal = FALSE)
pwise_counts_table_filter <- psych::describe(R2D_pairwise_counts_filter)
```

Table S5: Pairwise admins for subset of 70 items

	mean	sd	median	min	max	range
q_16294	619.39	24.95	617	567	682	115
q_16065	619.00	24.28	617	562	682	120
q_16057	604.35	25.74	603	559	691	132
q_16279	608.64	25.02	613	559	669	110
q_16235	610.01	26.04	610	558	669	111
q_16069	608.23	21.29	608	558	672	114
q_16230	609.49	27.53	609	555	675	120
q_16298	606.17	25.80	603	555	672	117
q_16195	615.48	24.99	619	554	685	131
q_16292	600.70	25.05	598	553	662	109
q_16056	608.09	23.49	607	552	677	125
q_16026	604.23	23.75	602	551	659	108
q_16023	608.83	22.86	609	550	657	107
q_16050	604.10	25.74	600	550	672	122
q_16086	596.45	24.23	592	549	675	126
q_16225	602.61	30.27	605	548	670	122
q_16009	597.88	22.51	597	548	646	98
q_16118	596.00	21.88	597	548	660	112
q_16138	596.72	25.15	594	548	651	103
q_16125	596.94	24.69	600	547	651	104
q_16042	609.19	28.37	611	546	681	135
q_16221	599.81	21.37	599	546	652	106
q_16061	597.32	23.40	596	546	657	111
q_16053	594.55	21.50	595	546	638	92
q_16087	612.61	23.86	616	546	671	125
q_16285	593.29	24.54	593	546	652	106
q_16035	591.59	25.71	592	546	640	94
q_16082	605.81	21.30	607	545	683	138
q_16281	602.28	25.01	601	545	668	123
q_16171	593.67	26.28	595	545	651	106
q_16016	600.06	26.56	601	545	665	120
q_16240	594.19	23.32	594	545	650	105
q_16140	600.54	24.62	602	544	652	108
q_16105	590.03	24.83	590	543	659	116
q_16258	600.80	26.06	602	541	667	126
q_16272	593.75	23.02	595	541	662	121
q_16193	592.51	23.15	592	541	652	111
q_16232	603.81	27.48	604	540	670	130
q_16001	588.29	22.65	587	538	643	105
q_16247	599.01	26.04	598	538	667	129
q_16287	587.75	25.88	586	538	655	117
q_16253	598.42	22.46	600	537	655	118
q_16085	589.94	24.94	588	537	648	111
q_16101	590.81	25.32	590	536	649	113
q_16048	602.06	27.17	601	535	683	148
q_16202	585.29	24.36	582	535	644	109
q_16203	591.48	28.49	593	533	654	121
q_16269	591.58	24.92	590	533	648	115
q_16109	595.29	30.44	591	530	663	133
q_16112	600.20	24.06	599	530	648	118
q_16063	593.19	25.92	592	530	681	151
q_16265	601.28	25.64	602	529	655	126
q_16220	593.91	27.28	594	529	670	141
q_16055	602.97	24.60	605	527	648	121

Table S5: Pairwise admins for subset of 70 items *(continued)*

	mean	sd	median	min	max	range
q_16104	587.88	27.04	589	527	658	131
q_16211	585.43	25.32	582	527	636	109
q_16004	581.36	26.88	579	526	643	117
q_16150	603.45	27.53	602	526	685	159
q_16249	588.74	26.28	591	526	647	121
q_16283	583.61	20.82	581	526	635	109
q_16047	589.33	25.94	591	525	672	147
q_16099	591.59	27.82	594	525	650	125
q_16223	592.29	27.28	590	524	679	155
q_16146	587.32	25.29	585	524	640	116
q_16008	578.59	22.13	581	524	623	99
q_16021	607.48	28.44	612	523	691	168
q_16102	594.38	27.81	592	518	661	143
q_16147	588.12	26.46	588	517	645	128
q_16144	587.20	28.47	582	511	649	138
q_16204	576.51	27.27	579	511	639	128

```
R2D_pairwise_counts_filter_summary <- as.vector(R2D_pairwise_counts_filter
                                     [lower.tri(R2D_pairwise_counts_filter)])

psych::describe(R2D_pairwise_counts_filter_summary)
```

```
##    vars    n   mean    sd median trimmed   mad min max range skew kurtosis   se
## X1    1 2415 597.48 26.73    597  597.34 26.69 511 691   180 0.06     0.01 0.54
```

Figure S2. pairwise administrations across item pairs

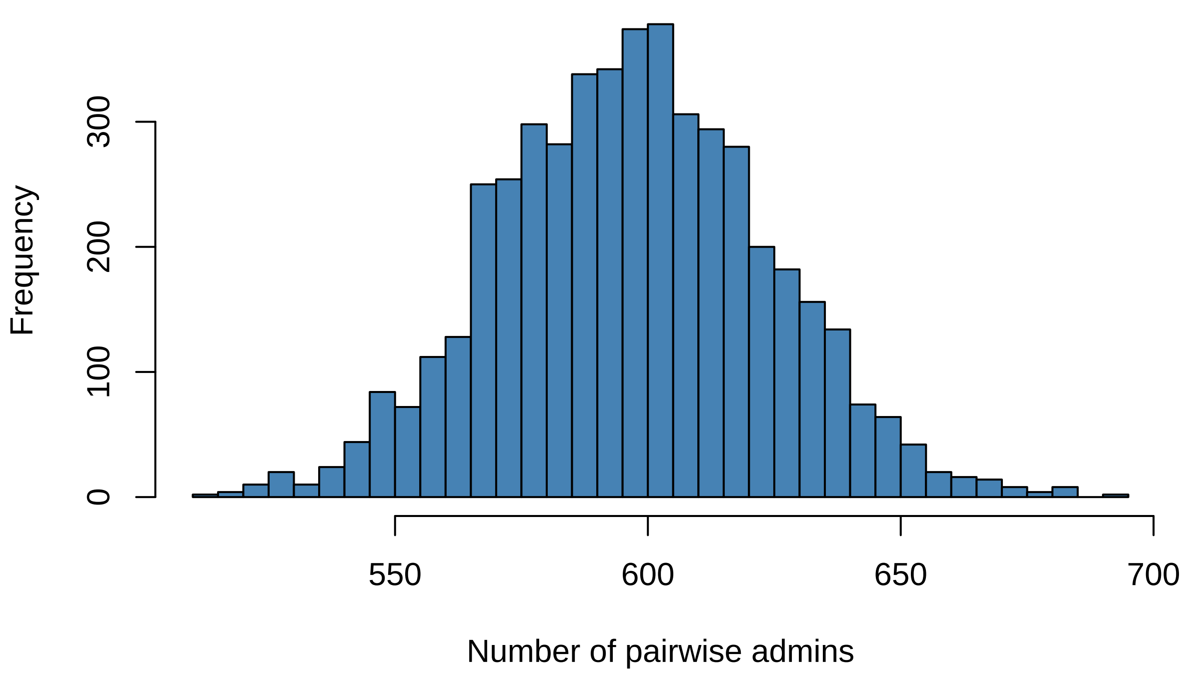

Factor structure and dimensionality

The following code was used to examine the factor structure and dimensionality of the 2D rotation items. Unidimensionality is assessed using the omega hierarchical (ω_h) coefficient, obtained using the 'omega()'

function from the psych package (Revelle, 2022). α and ω_t are used to evaluate internal consistency.

```
# Correlation matrix of subset of items
cor_matrix <- cor(Rot2D_subset, use = "pairwise")
cor_matrix_smoothed <- cor.smooth(cor_matrix)
```

The following images show the inter-item correlations arranged by general factor loading across factor solutions with 1 through 6 factors. Inspection of the plots suggests that there are up to four lower-order factors.

Figure S3. inter−item correlations for factor solutions with 1−6 factors

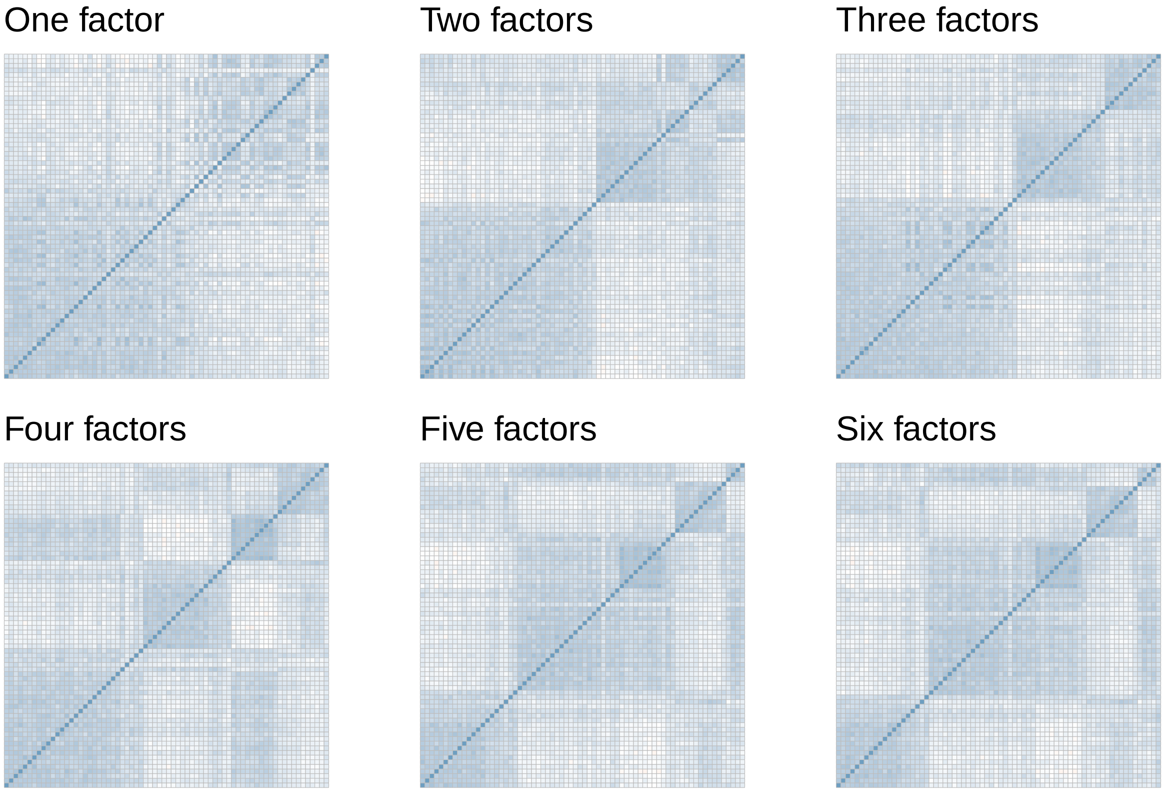

Next, the omega function is run for the one factor and four factor solutions to determine general factor saturation, indicated by ω_h and internal consistency (indicated by ω_t and α).

```
omega1 <- omega(cor_matrix_smoothed, plot = FALSE)
omega4 <- omega(cor_matrix_smoothed, nfactors = 4, plot = FALSE)

omega1$omega_h
```

```
## [1] 0.4749996
```

```
omega4$omega_h
```

```
## [1] 0.6645101
```

Omega hierarchical for the single-factor model ($\omega_h = 0.47$) suggests poor fit. Based on the correlation plots above and the improvement in unidimensionality when extracting four lower-order factors ($\omega_h = 0.66$), there appear to be several lower-order factors in the data. Table S6 shows the loadings of items by factor (see below for more description of the factor labels).

Table S6: Factor loadings

item	Rotate_both	Rotate_stimuli	Rotate_answer	Rotate_none	Communality	Uniqueness	Complexity
q_16109	0.76				0.51	0.49	1.15
q_16112	0.71				0.54	0.46	1.01
q_16147	0.68				0.45	0.55	1.01
q_16204	0.68				0.56	0.44	1.07
q_16102	0.66				0.47	0.53	1.04
q_16146	0.65				0.44	0.56	1.01
q_16104	0.64				0.47	0.53	1.04
q_16253	0.64				0.52	0.48	1.07
q_16021	0.64				0.54	0.46	1.15
q_16211	0.64				0.48	0.52	1.05
q_16099	0.64				0.42	0.58	1.00
q_16057	0.64				0.45	0.55	1.08
q_16001	0.62				0.50	0.50	1.13
q_16202	0.62				0.52	0.48	1.12
q_16247	0.61				0.44	0.56	1.07
q_16230	0.6				0.42	0.58	1.05
q_16225	0.6				0.41	0.60	1.05
q_16138	0.6				0.45	0.55	1.07
q_16004	0.59				0.50	0.50	1.26
q_16101	0.58				0.45	0.55	1.11
q_16047	0.51	0.22			0.42	0.58	1.56
q_16269	0.49				0.44	0.56	1.44
q_16249	0.48				0.43	0.57	1.40
q_16086	0.44				0.36	0.64	1.51
q_16035	0.43		0.26		0.42	0.58	1.99
q_16292	0.42	0.34			0.40	0.60	2.51
q_16144	0.41				0.19	0.81	1.06
q_16085	0.4	0.2			0.29	0.71	1.92
q_16023	0.3	0.3			0.38	0.62	2.71
q_16118	0.3	0.21	0.21		0.31	0.69	2.68
q_16240		0.76			0.58	0.42	1.02
q_16220		0.74			0.50	0.50	1.14
q_16087		0.71			0.51	0.49	1.01
q_16008		0.7			0.51	0.49	1.07
q_16042		0.7			0.54	0.46	1.15
q_16140		0.69			0.47	0.53	1.07
q_16258		0.68			0.49	0.51	1.04

Table S6: Factor loadings *(continued)*

item	Rotate_both	Rotate_stimuli	Rotate_answer	Rotate_none	Communality	Uniqueness	Complexity
q_16272		0.65			0.46	0.54	1.06
q_16150		0.65			0.48	0.52	1.10
q_16279		0.65			0.50	0.50	1.16
q_16221		0.64			0.52	0.48	1.16
q_16026		0.64			0.51	0.49	1.09
q_16298		0.64			0.43	0.57	1.10
q_16203		0.6			0.48	0.52	1.29
q_16048	0.31	0.46			0.37	0.63	1.95
q_16016	0.31	0.45			0.41	0.59	1.86
q_16223	0.32	0.44			0.35	0.65	1.91
q_16171		0.41			0.27	0.73	1.32
q_16265		0.37	0.31	0.34	0.42	0.58	3.56
q_16294			0.79		0.69	0.31	1.03
q_16235			0.76		0.58	0.42	1.09
q_16195			0.73		0.52	0.48	1.04
q_16056			0.67		0.58	0.42	1.11
q_16082			0.65		0.55	0.45	1.08
q_16287			0.65		0.60	0.40	1.13
q_16283	0.22		0.63		0.67	0.33	1.43
q_16050			0.6		0.57	0.43	1.35
q_16063	0.21		0.57		0.61	0.39	1.71
q_16055			0.56		0.42	0.58	1.16
q_16285				0.78	0.62	0.38	1.05
q_16105				0.76	0.58	0.42	1.15
q_16125				0.7	0.51	0.49	1.02
q_16069				0.69	0.51	0.49	1.03
q_16061				0.67	0.47	0.53	1.03
q_16053		0.25		0.63	0.53	0.47	1.34
q_16065		0.24		0.61	0.53	0.47	1.41
q_16232		0.24		0.56	0.45	0.55	1.41
q_16009		0.27		0.55	0.47	0.53	1.65
q_16281		0.25		0.54	0.46	0.54	1.76
q_16193			0.28	0.51	0.45	0.55	1.60

These lower-order factors appear to reflect differences in the type of mental rotation demanded by the items. Specifically, whether items require mental rotation of the stimuli, the answer option, both, or neither (i.e., the item does not require any mental rotation and merely requires participants to identify a missing piece of the stimuli). These factors represent all possible combinations of mental rotation of the stimuli and/or the answer option. An example of each of these four item types is shown below:

- **Factor 1**: Rotation of stimuli and of correct response option

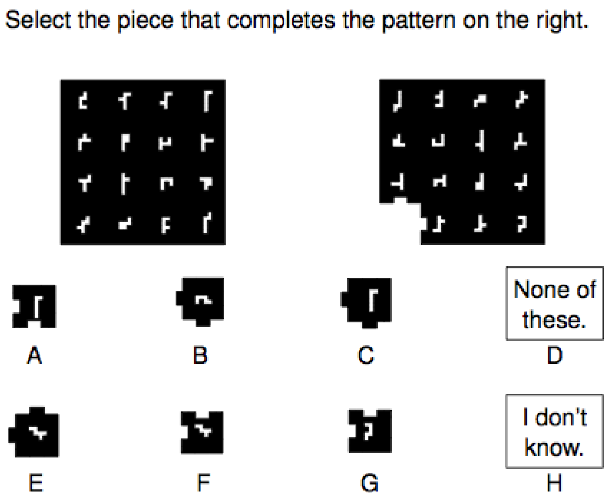

Figure S4. Example item for factor 1 ("rotate both")

- **Factor 2**: No rotation of stimuli, but requires rotation of correct response option

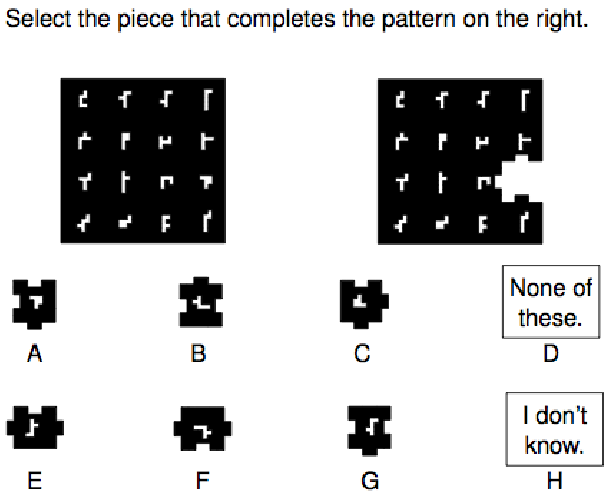

Figure S5. Example item for factor 2 ("rotate answer")

- **Factor 3**: Rotation of stimuli, no rotation of correct response option (i.e., answer option fits in missing puzzle piece without rotation).

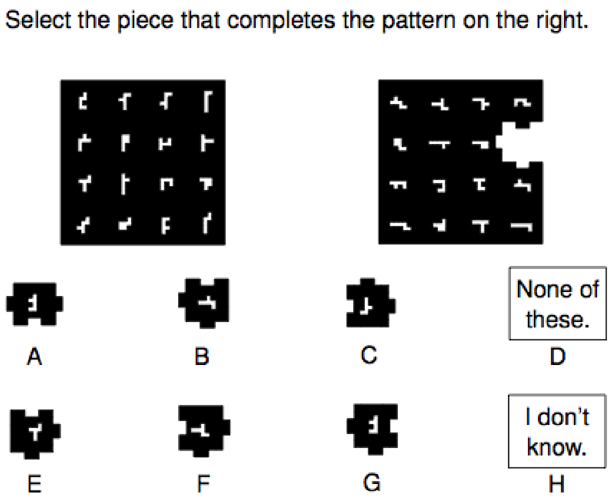

Figure S6. Example item for factor 3 ("rotate stimuli")

- **Factor 4**: No rotation

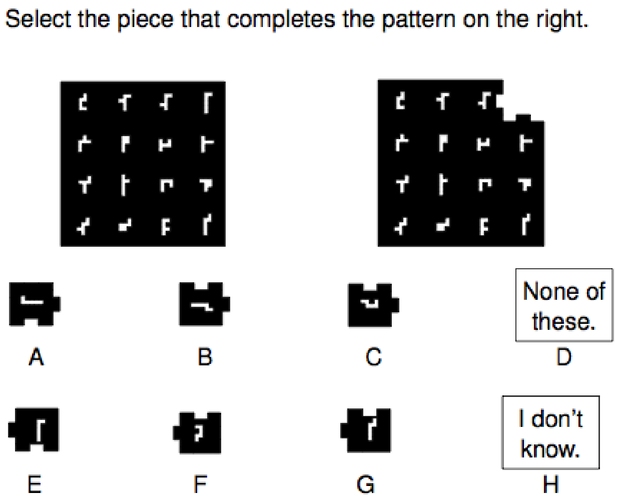

Figure S7. Example item for factor 4 ("rotate none")

Recall that omega hierarchical for the single-factor model suggests poor unidimensionality ($\omega_h = 0.47$). When extracting four lower-order factors, unidimensionality improves ($\omega_h = 0.66$), though it remains lower than is optimal for item response theory based scoring.

Items with primary loadings onto the fourth lower-order factor (example shown in figure S7) do not appear to require mental rotation. That is, participants do not need to rotate either the stimuli or the answer option to arrive at the correct answer and only need to be able to identify the puzzle piece that is missing from the stimuli (e.g., option "E" for the item in figure S7). We suspected that these items may be decreasing the unidimensionality of the scale–which is designed to measure mental rotation ability–and decided to remove these items and re-evaluate the factor structure of the items. After removing these 12 items, unidimensionality improved to an acceptable level ($\omega_h = 0.77$). After establishing adequate unidimensionality, we proceeded with IRT scoring.

IRT analyses of the items

First, IRT scores were generated for the general 2D rotation factor. All items from the pool were retained except for those loading onto the lower-order factor which did not require any mental rotation to solve. This left a total of 58 items. We also generate IRT scores for the four lower-order factors, including the ability to solve items that did not require rotation (i.e., the pattern-matching items).

Item parameters for general factor

Below, we remove items that do not require mental rotation and generate scores for general 2D rotation ability.

```
# Subsetting the data for unidimensional IRT scoring (remove items without rotation)
Rot2D_filtered <- Rot2D_subset %>%
  dplyr::select(-c("q_16009","q_16053", "q_16061", "q_16065", "q_16069",
  "q_16105", "q_16125", "q_16193", "q_16232", "q_16265", "q_16281", "q_16285"))

dim(Rot2D_filtered)
```

```
## [1] 1020195      58
```

```
IRT_model_unidimensional <- mirt(Rot2D_filtered, 1, itemtype = "2PL",
                            constrain = NULL)

save(IRT_model_unidimensional, file = "IRT_model_unidimensional.rdata")
```

```
# Item parameters for the general factor
IRT_model_unidimensional_parameters <- coef(IRT_model_unidimensional,
                                        IRTpars=TRUE, simplify = TRUE)

R2Dparameters <- IRT_model_unidimensional_parameters$items[,1:2]
R2Dparameters <- as.data.frame(R2Dparameters) %>%
                 dplyr::mutate(item = rownames(.)) %>%
                 relocate(item)
```

Descriptive statistics for the item parameters:

```
# Item discrimination
psych::describe(R2Dparameters$a)
```

```
##    vars  n mean   sd median trimmed  mad  min   max range skew kurtosis  se
## X1    1 58 2.03 2.28   1.72    1.74 0.62 0.89 18.63 17.74 6.66    45.68 0.3
```

```
# Item difficulty
psych::describe(R2Dparameters$b)
```

```
##    vars  n mean   sd median trimmed  mad  min  max range  skew kurtosis   se
## X1    1 58 0.35 0.69   0.43    0.35 0.77 -0.8 1.97  2.77 -0.02       -1 0.09
```

Plotting item probability functions and test information function

Figure S8. Item probability functions

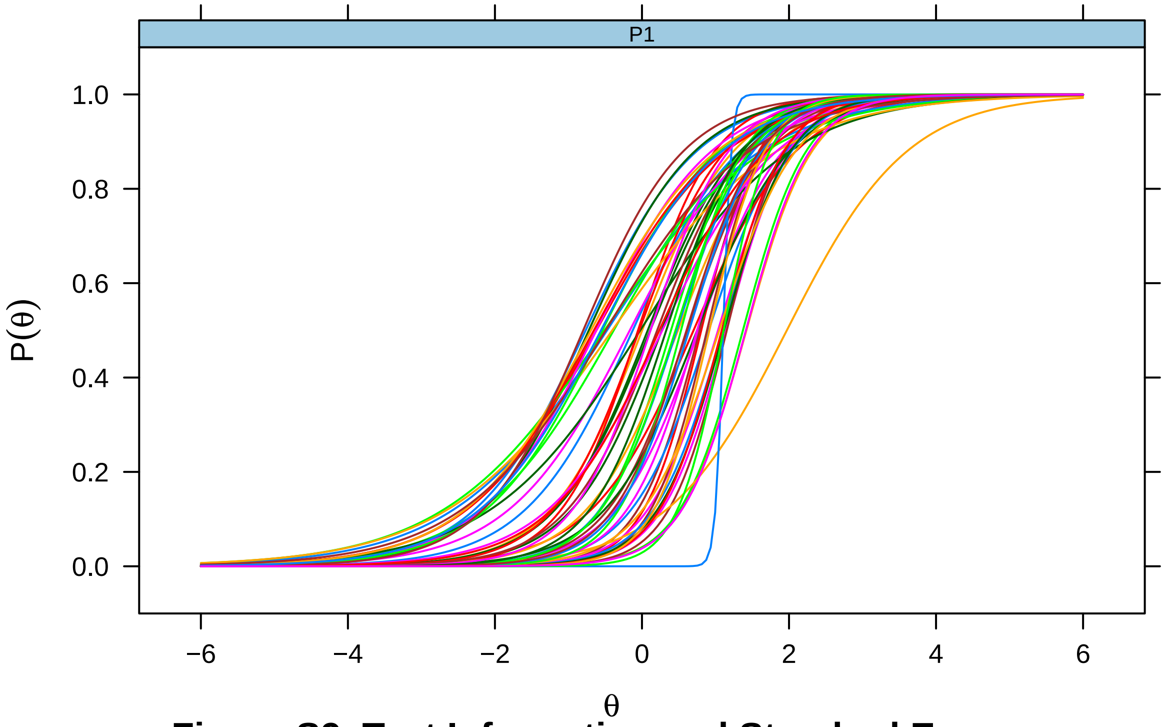

Figure S9. Test Information and Standard Errors

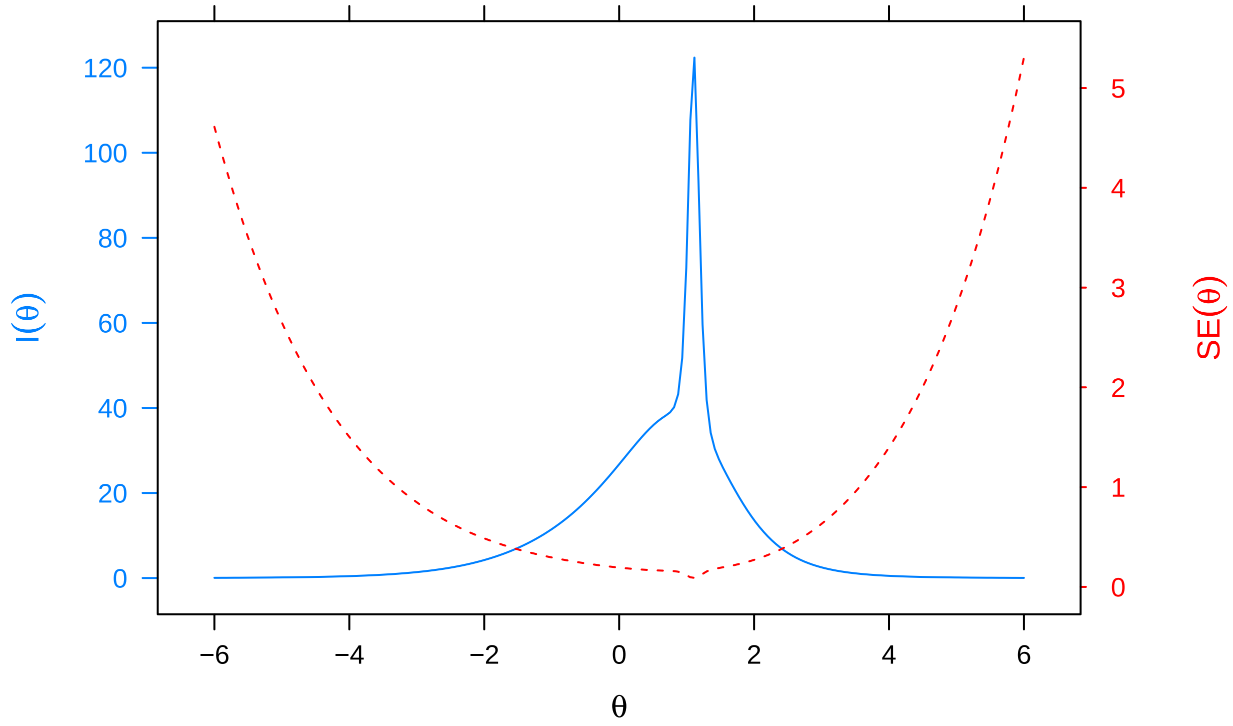

One of the items is interfering with the overall test. This item (q_16104) has a very high discrimination value. It is shown below and highlighted in the table with IRT parameters for each item. Note that this is also the most difficult item in the set (lowest mean with a proportion of .12 for correct responses), and that

it has a challenging distractor response option (see Figure S10).

Table S7: Item descriptives and IRT parameters, in descending order of item discrimination (potentially problematic items highlighted)

item	mean	sd	N	discrimination	difficulty
q_16104	0.12	0.33	23,106	18.63	1.11
q_16204	0.17	0.37	22,651	3.17	1.11
q_16202	0.23	0.42	22,677	2.65	0.92
q_16112	0.24	0.42	23,107	2.60	0.88
q_16253	0.26	0.44	23,627	2.54	0.81
q_16004	0.17	0.38	23,241	2.52	1.15
q_16021	0.27	0.44	23,590	2.36	0.79
q_16211	0.20	0.40	22,808	2.35	1.09
q_16001	0.35	0.48	22,795	2.35	0.53
q_16230	0.14	0.35	23,406	2.31	1.37
q_16099	0.13	0.34	22,517	2.29	1.41
q_16101	0.18	0.39	23,557	2.28	1.15
q_16102	0.13	0.34	23,438	2.23	1.42
q_16138	0.20	0.40	22,822	2.20	1.12
q_16147	0.20	0.40	22,899	2.12	1.10
q_16269	0.29	0.45	22,863	2.09	0.75
q_16109	0.21	0.41	23,388	2.08	1.03
q_16057	0.40	0.49	23,617	2.06	0.39
q_16247	0.33	0.47	22,769	2.02	0.64
q_16035	0.34	0.47	22,640	2.00	0.61
q_16249	0.38	0.49	22,922	1.96	0.46
q_16146	0.22	0.42	22,798	1.92	1.05
q_16047	0.38	0.48	22,995	1.91	0.47
q_16283	0.41	0.49	22,759	1.91	0.33
q_16023	0.52	0.50	23,502	1.90	-0.05
q_16225	0.26	0.44	23,584	1.89	0.91
q_16287	0.47	0.50	22,760	1.87	0.12
q_16294	0.33	0.47	23,399	1.84	0.62
q_16086	0.30	0.46	23,741	1.74	0.78
q_16016	0.52	0.50	23,053	1.70	-0.04
q_16063	0.45	0.50	23,024	1.63	0.21
q_16235	0.43	0.50	23,713	1.63	0.27
q_16056	0.51	0.50	23,619	1.60	0.00
q_16050	0.37	0.48	23,318	1.60	0.49
q_16118	0.31	0.46	22,962	1.58	0.75
q_16292	0.48	0.50	23,370	1.58	0.08
q_16082	0.48	0.50	23,507	1.54	0.11
q_16240	0.71	0.46	22,785	1.46	-0.80
q_16195	0.32	0.47	23,344	1.43	0.70
q_16279	0.68	0.47	23,165	1.38	-0.72
q_16048	0.54	0.50	23,388	1.36	-0.13
q_16085	0.45	0.50	22,692	1.36	0.24
q_16026	0.68	0.46	23,839	1.32	-0.77
q_16223	0.44	0.50	22,971	1.28	0.28
q_16258	0.65	0.48	23,095	1.27	-0.62
q_16150	0.63	0.48	23,032	1.26	-0.54
q_16144	0.13	0.33	23,155	1.22	1.97
q_16055	0.54	0.50	23,180	1.21	-0.17
q_16008	0.63	0.48	22,537	1.21	-0.56
q_16203	0.66	0.48	22,938	1.16	-0.69
q_16087	0.65	0.48	22,953	1.14	-0.65
q_16221	0.64	0.48	23,157	1.13	-0.62
q_16298	0.59	0.49	23,283	1.10	-0.38
q_16220	0.61	0.49	22,901	1.02	-0.49
q_16171	0.51	0.50	23,063	0.98	-0.02
q_16042	0.60	0.49	23,208	0.95	-0.48
q_16140	0.60	0.49	23,143	0.91	-0.51
q_16272	0.58	0.49	23,048	0.89	-0.40

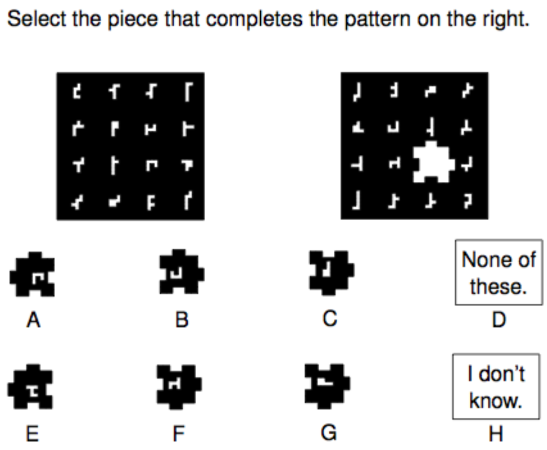

Figure S10. One item with an unusually high discrimination value

Dropping item and re-running the analyses:

Given the unusual difficulty of this item, we decided to drop it and re-run the analyses to see whether this improves the psychometric properties of the test overall. In fact, it does (see below), so the item has been permanently deprecated.

```
# Dropping 16104
Rot2D_filtered2 <- Rot2D_filtered %>% dplyr::select(-q_16104)
```

```
# Re-running the IRT model
IRT_model_unidimensional2 <- mirt(Rot2D_filtered2, 1, itemtype = "2PL",
                             constrain = NULL)

save(IRT_model_unidimensional2, file = "IRT_model_unidimensional2.rdata")
```

```
load("/Users/kmather3/Desktop/Projects/2D_Rotation/IRT_model_unidimensional2.rdata")

# Item parameters
IRT_model_unidimensional_parameters2 <- coef(IRT_model_unidimensional2,
                                        IRTpars=TRUE, simplify = TRUE)

R2Dparameters2 <- IRT_model_unidimensional_parameters2$items[,1:2]
R2Dparameters2 <- as.data.frame(R2Dparameters2) %>%
                 dplyr::mutate(item = rownames(.)) %>%
                 relocate(item)
```

Plotting item probability functions and test information

Figure S11. Item Probability Functions

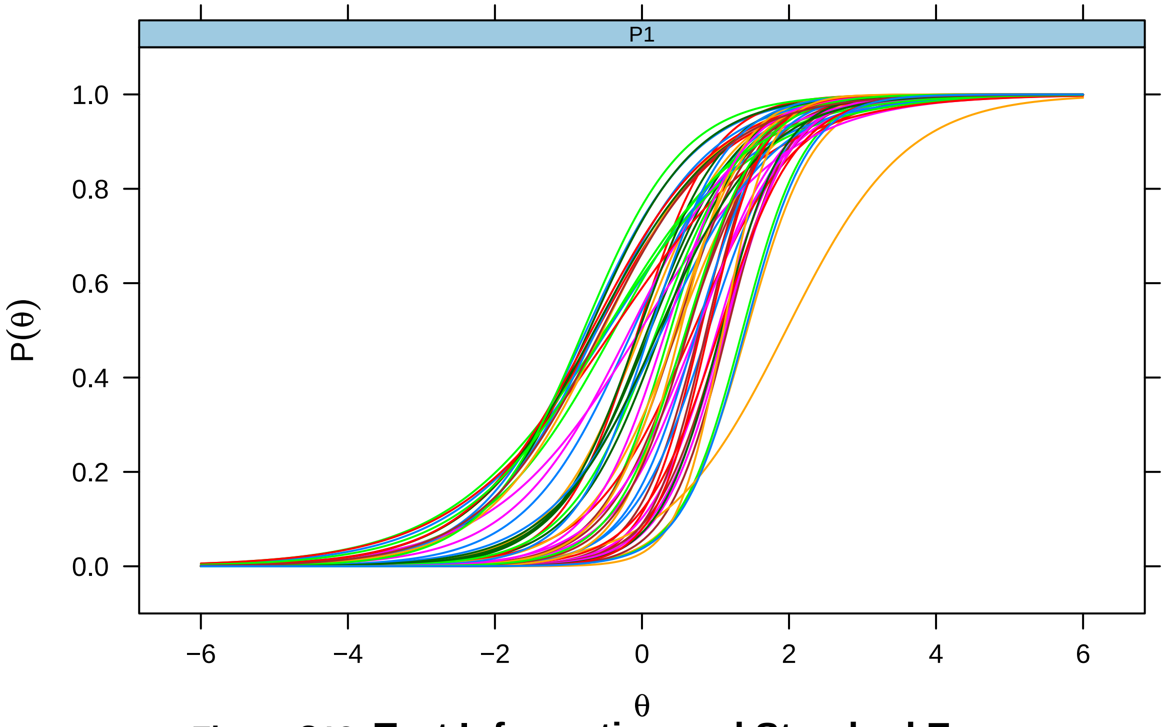

Figure S12. Test Information and Standard Errors

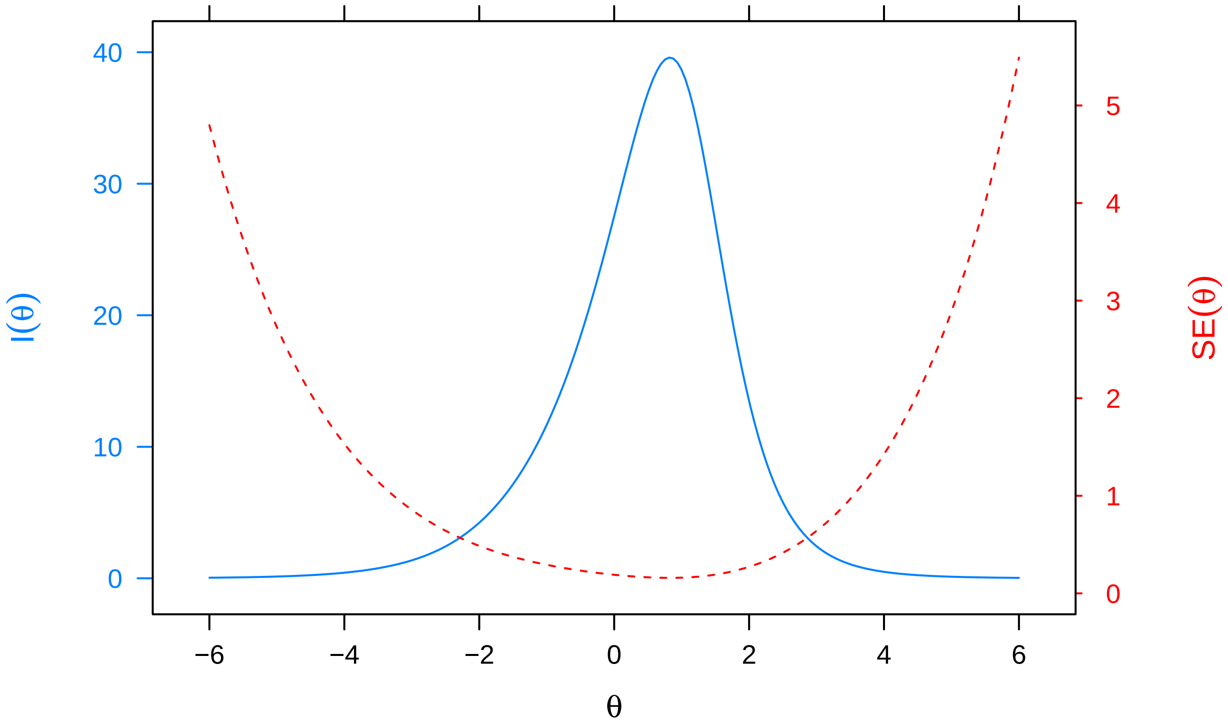

One additional item (shown in Figure S13) stood out for having a relatively high difficulty level. Similar to item 16104, which was dropped from the final scale, this item has a challenging distractor option. We decided to retain this item because analyses suggest that it does not interfere with the psychometric properties of the

overall scale.

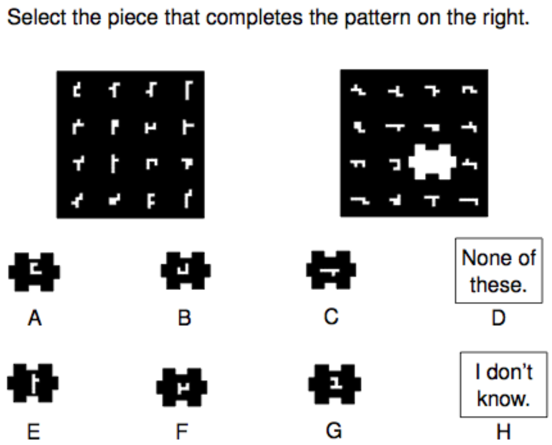

Figure S13. One other item (item 16144) had a fairly high difficulty level and a similar but less challenging distractor option

Table S8: Item descriptives and IRT parameters, in descending order of item discrimination

item	mean	sd	N	discrimination	difficulty
q_16204	0.17	0.37	22,651	3.21	1.10
q_16202	0.23	0.42	22,677	2.72	0.91
q_16112	0.24	0.42	23,107	2.65	0.88
q_16253	0.26	0.44	23,627	2.60	0.80
q_16004	0.17	0.38	23,241	2.47	1.16
q_16001	0.35	0.48	22,795	2.44	0.52
q_16211	0.20	0.40	22,808	2.39	1.08
q_16021	0.27	0.44	23,590	2.38	0.78
q_16230	0.14	0.35	23,406	2.33	1.37
q_16099	0.13	0.34	22,517	2.32	1.40
q_16101	0.18	0.39	23,557	2.29	1.14
q_16138	0.20	0.40	22,822	2.21	1.11
q_16102	0.13	0.34	23,438	2.19	1.43
q_16147	0.20	0.40	22,899	2.15	1.09
q_16109	0.21	0.41	23,388	2.12	1.03
q_16057	0.40	0.49	23,617	2.12	0.39
q_16269	0.29	0.45	22,863	2.11	0.74
q_16247	0.33	0.47	22,769	2.08	0.63
q_16035	0.34	0.47	22,640	2.03	0.60
q_16249	0.38	0.49	22,922	2.02	0.45
q_16023	0.52	0.50	23,502	1.94	-0.05
q_16047	0.38	0.48	22,995	1.93	0.47
q_16283	0.41	0.49	22,759	1.93	0.33
q_16287	0.47	0.50	22,760	1.93	0.11
q_16225	0.26	0.44	23,584	1.91	0.90
q_16146	0.22	0.42	22,798	1.91	1.05
q_16294	0.33	0.47	23,399	1.85	0.61
q_16086	0.30	0.46	23,741	1.77	0.77
q_16016	0.52	0.50	23,053	1.75	-0.04
q_16235	0.43	0.50	23,713	1.66	0.26
q_16063	0.45	0.50	23,024	1.65	0.20
q_16292	0.48	0.50	23,370	1.63	0.08
q_16050	0.37	0.48	23,318	1.62	0.48
q_16056	0.51	0.50	23,619	1.60	0.00
q_16118	0.31	0.46	22,962	1.59	0.74
q_16082	0.48	0.50	23,507	1.55	0.11
q_16240	0.71	0.46	22,785	1.48	-0.79
q_16195	0.32	0.47	23,344	1.44	0.70
q_16279	0.68	0.47	23,165	1.39	-0.72
q_16085	0.45	0.50	22,692	1.39	0.23
q_16048	0.54	0.50	23,388	1.37	-0.13
q_16026	0.68	0.46	23,839	1.34	-0.76
q_16223	0.44	0.50	22,971	1.30	0.27
q_16258	0.65	0.48	23,095	1.28	-0.62
q_16150	0.63	0.48	23,032	1.28	-0.53
q_16055	0.54	0.50	23,180	1.24	-0.17
q_16144	0.13	0.33	23,155	1.23	1.96
q_16008	0.63	0.48	22,537	1.22	-0.55
q_16203	0.66	0.48	22,938	1.18	-0.69
q_16087	0.65	0.48	22,953	1.17	-0.65
q_16221	0.64	0.48	23,157	1.15	-0.61
q_16298	0.59	0.49	23,283	1.12	-0.38
q_16220	0.61	0.49	22,901	1.03	-0.49
q_16171	0.51	0.50	23,063	0.99	-0.02
q_16042	0.60	0.49	23,208	0.98	-0.47
q_16140	0.60	0.49	23,143	0.93	-0.50
q_16272	0.58	0.49	23,0[illegible]8	0.91	-0.40

```r
plot(IRT_model_unidimensional2, type = 'trace', facet_items = TRUE,
     main="Figure 14. Item Probability Functions")
```

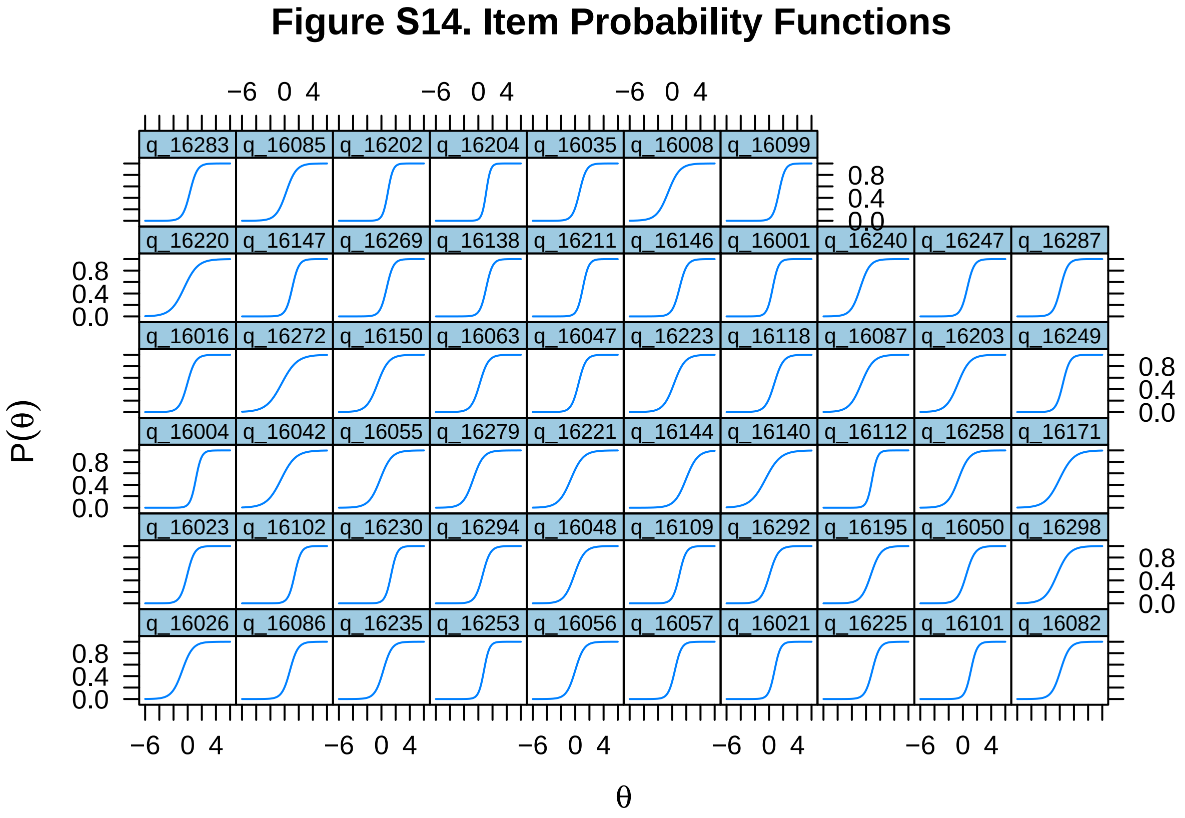

Descriptive statistics for the item parameters:

```r
# Item discrimination
psych::describe(R2Dparameters2$a)
```

```
##    vars  n mean   sd median trimmed mad  min  max range skew kurtosis   se
## X1    1 57 1.76 0.53   1.75    1.74 0.6 0.91 3.21   2.3 0.34    -0.59 0.07
```

```r
# Item difficulty
psych::describe(R2Dparameters2$b)
```

```
##    vars  n mean   sd median trimmed  mad   min  max range skew kurtosis   se
## X1    1 57 0.34 0.68   0.39    0.33 0.76 -0.79 1.96  2.75 0.02    -0.98 0.09
```

Lower-order factors

When scoring the lower-order factors, the full set of items is used (i.e., items requiring no rotation are not removed because these items form one of the lower-order factors). Item labels are obtained from the omega function output, and then the unidimensionality of each subfactor is evaluated.

```r
# Item labels from the 'omega()' function output
F1_items <- omega4$model$lavaan[2]
F1_items <- unlist(stringr::str_extract_all(F1_items, "q_[:digit:]{5}"))
F2_items <- omega4$model$lavaan[3]
F2_items <- unlist(stringr::str_extract_all(F2_items, "q_[:digit:]{5}"))
F3_items <- omega4$model$lavaan[4]
F3_items <- unlist(stringr::str_extract_all(F3_items, "q_[:digit:]{5}"))
F4_items <- omega4$model$lavaan[5]
F4_items <- unlist(stringr::str_extract_all(F4_items, "q_[:digit:]{5}"))

# Unidimensionality of each subfactor

# Factor 1
cor_matrixF1<- cor(Rot2D_subset[,F1_items], use = "pairwise")
cor_matrix_smoothedF1 <- cor.smooth(cor_matrixF1)
omega_F1<- omega(cor_matrix_smoothedF1, plot = FALSE)
round(omega_F1$omega_h,2)
```

```
## [1] 0.92
```

```r
# Factor 2
cor_matrixF2<- cor(Rot2D_subset[,F2_items], use = "pairwise")
cor_matrix_smoothedF2 <- cor.smooth(cor_matrixF2)
omega_F2<- omega(cor_matrix_smoothedF2, plot = FALSE)
round(omega_F2$omega_h,2)
```

```
## [1] 0.88
```

```r
# Factor 3
cor_matrixF3<- cor(Rot2D_subset[,F3_items], use = "pairwise")
cor_matrix_smoothedF3 <- cor.smooth(cor_matrixF3)
omega_F3<- omega(cor_matrix_smoothedF3, plot = FALSE)
round(omega_F3$omega_h,2)
```

```
## [1] 0.81
```

```r
# Factor 4
cor_matrixF4 <- cor(Rot2D_subset[,F4_items], use = "pairwise")
cor_matrix_smoothedF4 <- cor.smooth(cor_matrixF4)
omega_F4 <- omega(cor_matrix_smoothedF4, plot = FALSE)
round(omega_F4$omega_h,2)
```

```
## [1] 0.84
```

Because each lower-order factor demonstrates sufficient unidimensionality, we obtain IRT scale scores for them below.

```r
# First factor: rotate both square and answer option
IRT_model_F1 <- mirt(Rot2D_subset[,F1_items], 1,
                     itemtype = "2PL",
                     constrain = NULL)

# Second factor: rotate square
IRT_model_F2 <- mirt(Rot2D_subset[,F2_items], 1,
                     itemtype = "2PL",
                     constrain = NULL)

# Third factor: no rotation
IRT_model_F3 <- mirt(Rot2D_subset[,F3_items], 1,
                     itemtype = "2PL",
                     constrain = NULL)

# Fourth factor: rotate answer option
IRT_model_F4 <- mirt(Rot2D_subset[,F4_items], 1,
                     itemtype = "2PL",
                     constrain = NULL)


save(IRT_model_F1, file = "IRT_model_F1.rdata")
save(IRT_model_F2, file = "IRT_model_F2.rdata")
save(IRT_model_F3, file = "IRT_model_F3.rdata")
save(IRT_model_F4, file = "IRT_model_F4.rdata")
```

```r
load("/Users/kmather3/Desktop/Projects/2D_Rotation/IRT_model_F1.rdata")
load("/Users/kmather3/Desktop/Projects/2D_Rotation/IRT_model_F2.rdata")
load("/Users/kmather3/Desktop/Projects/2D_Rotation/IRT_model_F3.rdata")
load("/Users/kmather3/Desktop/Projects/2D_Rotation/IRT_model_F4.rdata")

# First factor
IRT_model_F1_parameters <- coef(IRT_model_F1, IRTpars=TRUE, simplify = TRUE)
R2Dparameters_F1 <- IRT_model_F1_parameters$items[,1:2]

# Second factor
IRT_model_F2_parameters <- coef(IRT_model_F2, IRTpars=TRUE, simplify = TRUE)
R2Dparameters_F2 <- IRT_model_F2_parameters$items[,1:2]

# Third factor
IRT_model_F3_parameters <- coef(IRT_model_F3, IRTpars=TRUE, simplify = TRUE)
R2Dparameters_F3 <- IRT_model_F3_parameters$items[,1:2]

# fourth factor
IRT_model_F4_parameters <- coef(IRT_model_F4, IRTpars=TRUE, simplify = TRUE)
R2Dparameters_F4 <- IRT_model_F4_parameters$items[,1:2]
```

Plotting the test information for each item type

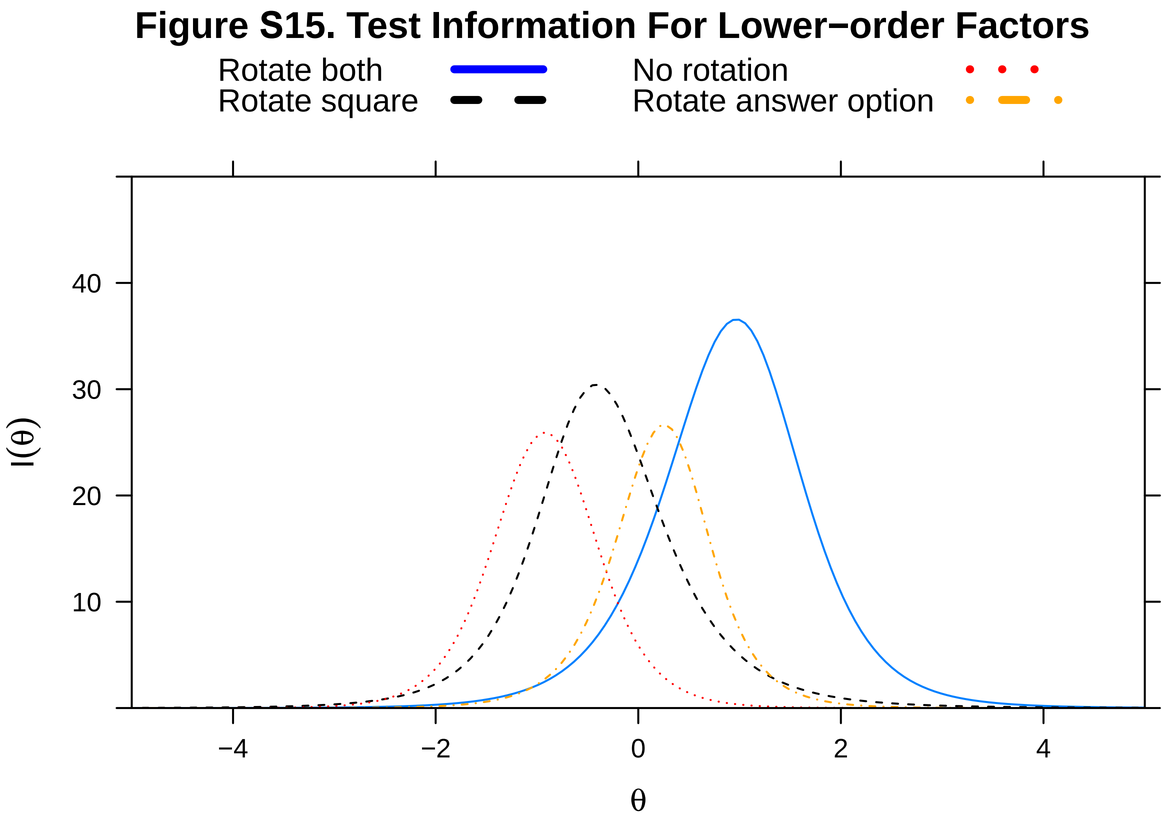

The code below was used to get IRT scores for general 2D rotation ability and the four lower-order factors.

```
ICARdata$R2D_IRT_general = fscores(IRT_model_unidimensional2)
ICARdata$R2D_IRT_Rotate_Both = fscores(IRT_model_F1)
ICARdata$R2D_IRT_Rotate_Square = fscores(IRT_model_F2)
ICARdata$R2D_IRT_Rotate_None = fscores(IRT_model_F3)
ICARdata$R2D_IRT_Rotate_Answer = fscores(IRT_model_F4)
```

Correlations between the lower-order 2D rotation abilities (based on IRT scores) are shown below. Correlations between the lower-order abilities were weak to moderate. The strongest of these relationships was between the ability to rotate the answer option only and the ability to rotate both the stimuli and the answer option ($r = .39$). All other correlations were under .30. All 95% confidence intervals were +/- $|.012|$.

```
R2D_cols <- c("Rotate stimuli and answer", "Rotate stimuli only",
              "No rotation", "Rotate answer only")

R2D_abilities <- ICARdata %>%
  dplyr::select(R2D_IRT_Rotate_Both:R2D_IRT_Rotate_Answer) %>%
  setNames(R2D_cols)
```

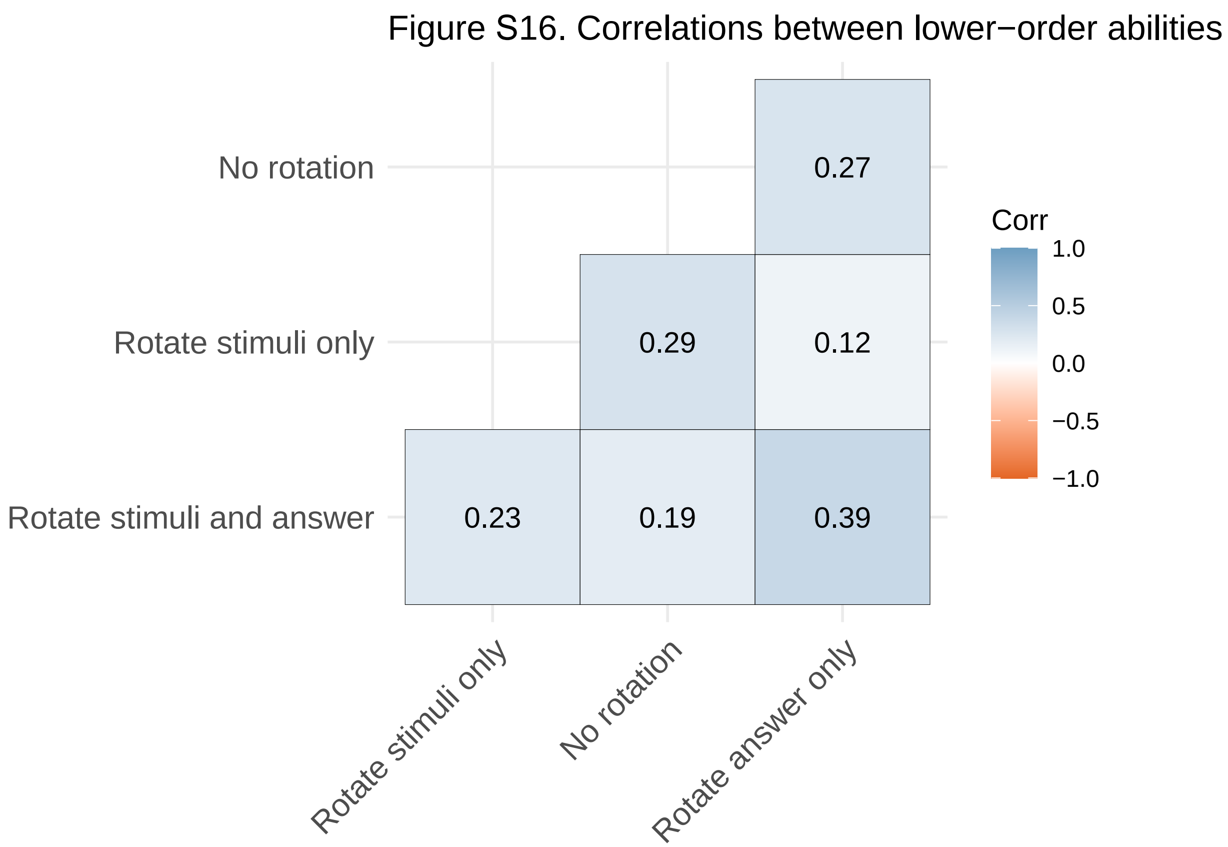

Correlations between 2D rotation and other individual differences

The following plots show the correlations between 2D rotation ability and a variety of personality traits and cognitive abilities.

Cogitive abilities

```
ability_cols <- c("2D-rotation", "Figural analogies", "Propositional reasoning",
                  "Letter-number series", "3D-rotation", "Matrix reasoning",
                  "Verbal reasoning", "Compound remote associates")


# t-scoring 2D rotation scores
ICARdata <- ICARdata %>%
  mutate(R2D_IRT_general = ((R2D_IRT_general - mean(R2D_IRT_general, na.rm=TRUE))/
  sd(R2D_IRT_general, na.rm=TRUE))*10+50)

abilities <- ICARdata %>%
  dplyr::select(R2D_IRT_general,
  ICAR_FA_IRT_score:ICAR_CRA_IRT_score) %>%
  setNames(ability_cols)
```

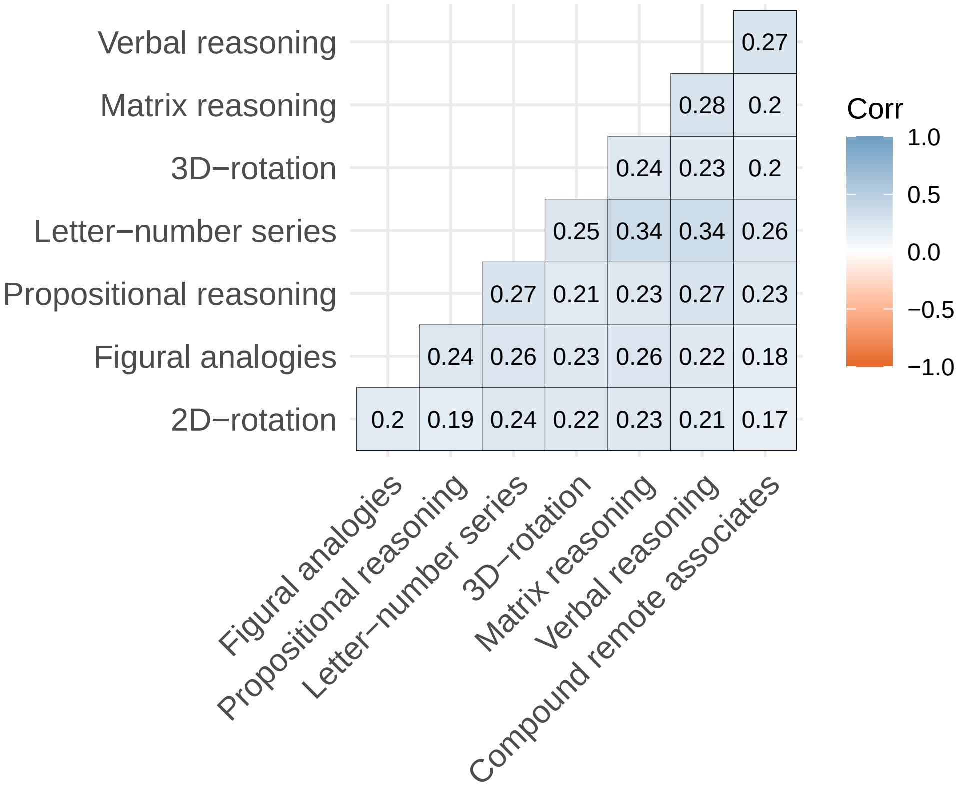

Figure S17. Correlations with other cognitive abilities

(all 95% confidence intervals +/- |.003|)

Temperament

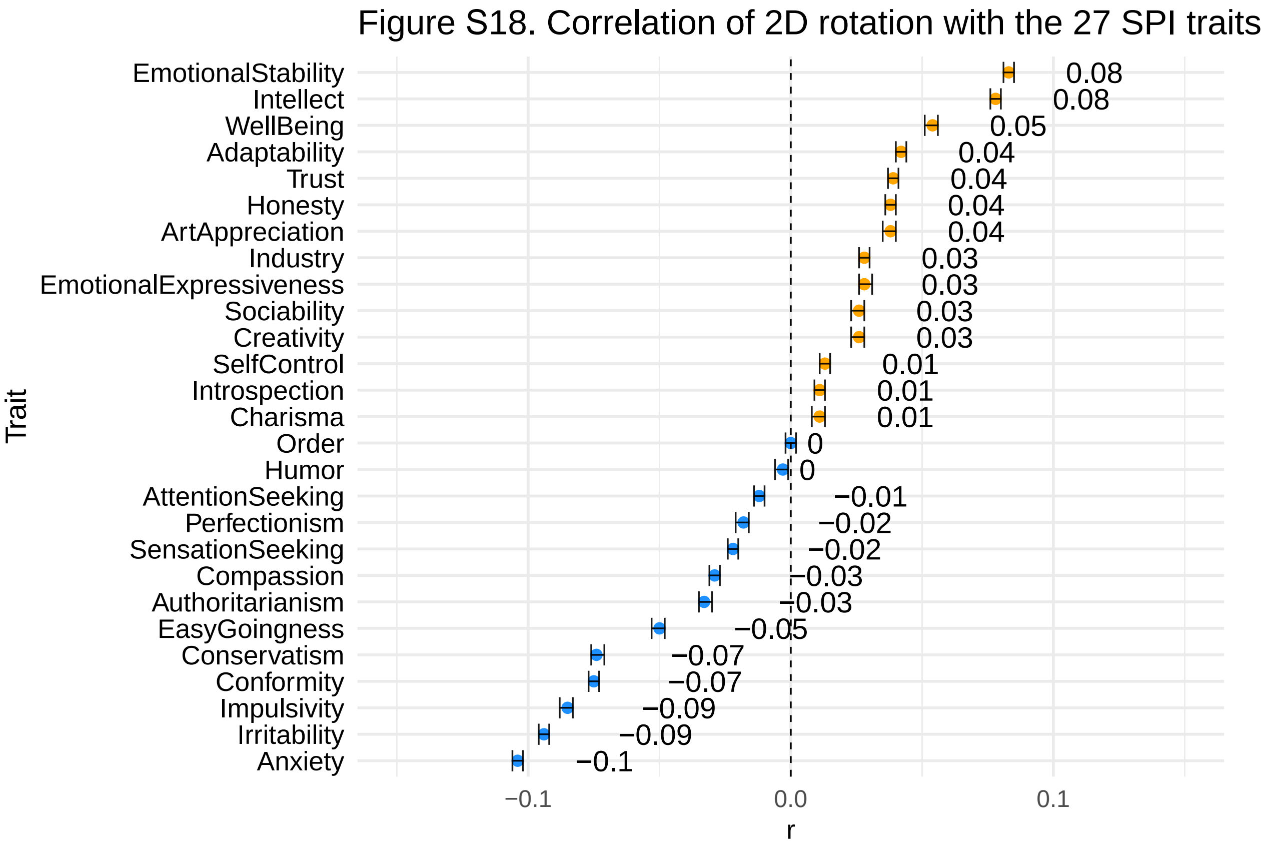

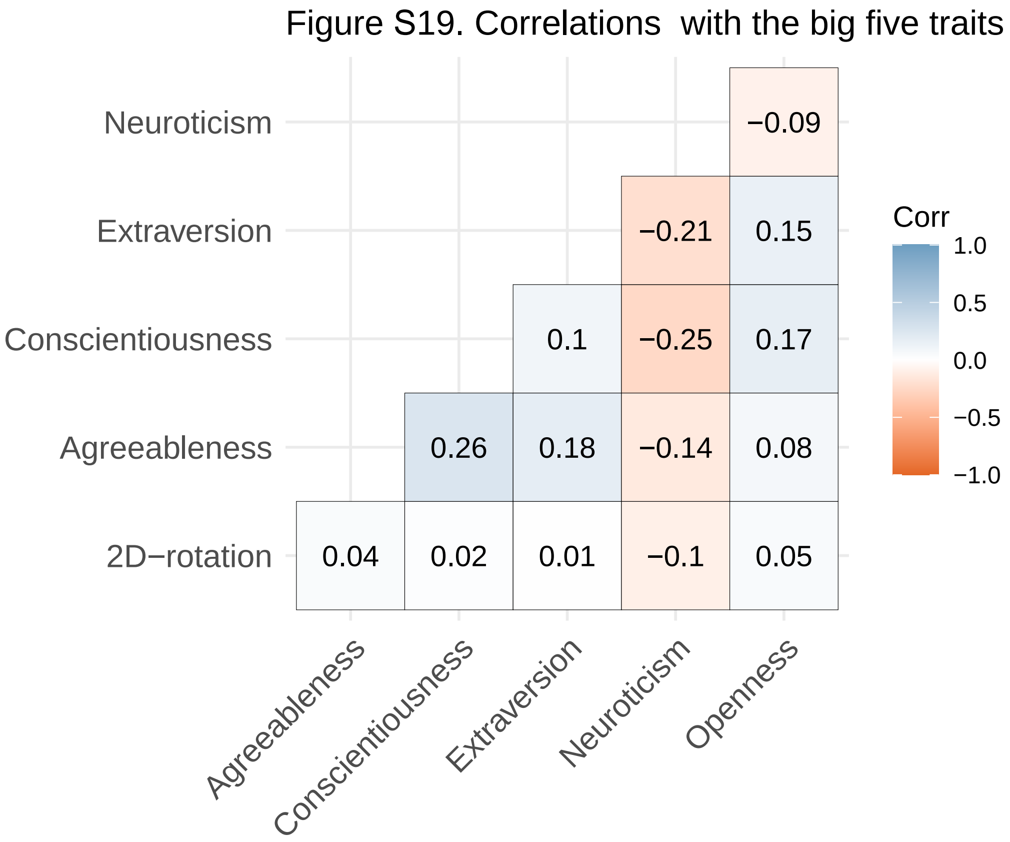

(All 95% Confidence Intervals +/- |.003|)

O*NET job characteristics

Next, we examine the job characteristics most strongly associated with 2D rotation ability. Job characteristics are described on the ONET (https://www.onetonline.org/) website and can be organized into the six themes outlined by the ONET content model shown in the figure below.

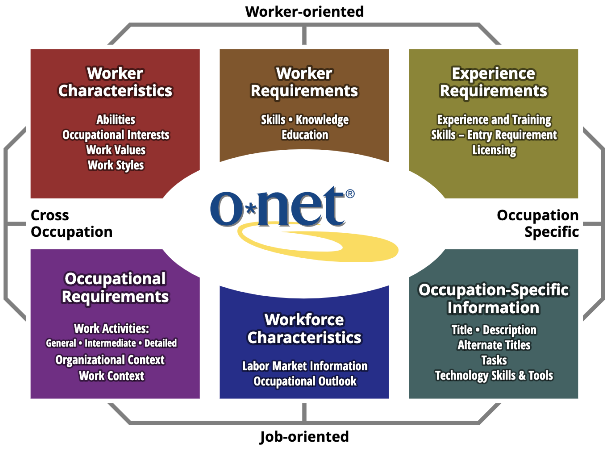

Figure S20. O*NET Content model

First, we load the data with job characteristics and O*NET occupation codes to merge with SAPA data. We then use the BISCUIT statistical learning technique (Elleman et al., 2020) to identify the job characteristics

most strongly associated with 2D rotation ability.

```
onet_data <- import(here("Data", "onet_char.csv"))
types <- import(here("Data", "onet_varnames_char_types.csv"))
CrosswalkTable <- import(here("Data", "PrestigeRtgsCrosswalk28apr2021.csv")) # File with Occupational codes

# Dropping "level" job characteristics (only "importance" ratings for the characteristics will be used)
no_redund_data <- onet_data %>% dplyr::select(-ends_with("_Level"))
# Drop some variables that had highly redundant pairs (r>.9) following discussion among raters
varsToDrop <- types %>% filter(drop == "1")
# This removes 7 additional items
no_redund_data <- no_redund_data[, !names(no_redund_data) %in% varsToDrop$char_name]

CrosswalkTable$`O*NET-SOC 2010 Code` <- str_replace_all(CrosswalkTable$`O*NET-SOC 2010 Code`, "[^[:alnum:]]", "")

# Getting 2D rotation scores, grouping by occupation, and getting averages
R2D_data <- ICARdata %>%
  select(R2D_IRT_general, occupation) %>%
  group_by(occupation) %>%
  dplyr::summarise(R2D_Score = mean(R2D_IRT_general, na.rm = TRUE),
  n = length(!is.na(R2D_IRT_general))) %>%
  dplyr::filter(n>=100) %>%
  mutate('SAPA Job Title' = occupation)

# Joining the O*NET and R2D data
R2D_OPR <- inner_join(R2D_data, CrosswalkTable, by = "SAPA Job Title") %>%
  rename("occ_code" = "O*NET-SOC 2010 Code") %>%
  mutate(occ_code = as.numeric(occ_code)) %>%
  select(occ_code, occupation, R2D_Score, n, "SAPA Job Title")

R2D_OPR_onet <- left_join(no_redund_data, R2D_OPR, by = "occ_code")
# remove duplicated rows
R2D_OPR_onet <- R2D_OPR_onet %>% distinct()
# Edit the _Imp labels
names(R2D_OPR_onet) = gsub("_Imp", "", names(R2D_OPR_onet))


# running the bestScales function
bs_all <- bestScales(R2D_OPR_onet[,c(2:240,242)], criteria = cs(R2D_Score),
         cut = .0, n.item = 100, n.iter = 10, folds = 10,
         overlap = FALSE, check = TRUE, impute = "none", digits = 2)

# Getting a data frame with the job characteristics
bs_model_R2D <- as.data.frame(bs_all$items$R2D_Score)
bs_model_R2D$mean.r <- round(bs_model_R2D$mean.r, 2)
bs_model_R2D$sd.r <- round(bs_model_R2D$sd.r, 2)
rownames(bs_model_R2D) <- gsub("_", " ", rownames(bs_model_R2D))
colnames(bs_model_R2D) <- c("Frequency", "Mean Correlation", "Standard Deviation")
```

Table S9: 40 Job Characteristics Most Strongly associated with 2D Rotation

	Frequency	Mean Correlation	Standard Deviation
**20 strongest positive associations**			
Engineering and Technology	10	0.56	0.01
Physics Knowledge	10	0.52	0.01
Investigative	10	0.52	0.01
Mathematical Reasoning	10	0.49	0.01
Science	10	0.49	0.01
Mathematics Knowledge	10	0.48	0.01
Mathematics Skills	10	0.48	0.01
Design	10	0.48	0.01
Technology Design	10	0.48	0.01
Analytical Thinking	10	0.46	0.01
Estimating the Quantifiable Characteristics of Products Events or Information	10	0.46	0.01
Systems Analysis	10	0.46	0.01
Information Ordering	10	0.46	0.01
Programming	10	0.45	0.01
Number Facility	10	0.45	0.01
Analyzing Data or Information	10	0.44	0.01
Category Flexibility	10	0.44	0.02
Operations Analysis	10	0.43	0.01
Complex Problem Solving	10	0.42	0.01
Visualization	10	0.42	0.01
**20 strongest negative associations**			
Frequency of Conflict Situations	9	-0.21	0.01
Therapy and Counseling	10	-0.21	0.01
Social Perceptiveness	10	-0.22	0.01
Physical Proximity	10	-0.22	0.02
Stress Tolerance	10	-0.23	0.01
Spend Time Making Repetitive Motions	10	-0.23	0.01
Clerical	10	-0.24	0.02
Deal With Physically Aggressive People	10	-0.27	0.01
Assisting and Caring for Others	10	-0.29	0.01
Contact With Others	10	-0.31	0.02
Service Orientation	10	-0.31	0.01
Social	10	-0.32	0.01
Deal With External Customers	10	-0.33	0.02
Relationships	10	-0.33	0.02
Concern for Others	10	-0.34	0.02
Social Orientation	10	-0.35	0.01
Self Control	10	-0.35	0.02
Customer and Personal Service	10	-0.39	0.02
Deal With Unpleasant or Angry People	10	-0.39	0.01
Performing for or Working Directly with the Public	10	-0.39	0.01

Occupations and Majors

Below, we examine the highest scoring college majors and occupations on 2D rotation ability in comparison with 3D rotation ability.

Occupations

```r
# Getting the highest scoring occupations
occupations <- ICARdata %>%
  dplyr::group_by(occupation) %>%
  dplyr::summarise(R2D_Score = mean(R2D_IRT_general, na.rm = TRUE),
  R3D_Score = mean(ICAR_R3D_IRT_score, na.rm = TRUE),
  n = length(!is.na(R2D_IRT_general))) %>%
  dplyr::mutate(R2D_Percentile = round(dplyr::percent_rank(R2D_Score),2),
  R3D_Percentile = round(dplyr::percent_rank(R3D_Score),2) # Percentiles
  )
```

Table S10: Highest scoring occupations on 2D rotation (in descending order of 2D rotation percentile)

occupation	N	R2D	R3D	Difference
Biologists	554	0.90	0.91	-0.01
Computer Systems Engineers/Architect	1351	0.89	0.95	-0.06
Mechanical Engineer	1034	0.89	0.92	-0.03
Network and Computer Systems Administrator	828	0.86	0.87	-0.01
Web Developer	1028	0.84	0.93	-0.09
Computer Software Engineer	4480	0.83	0.93	-0.10
Architect	1596	0.82	0.81	0.01
Electrical Engineers	923	0.82	0.86	-0.04
Engineering Manager	642	0.82	0.93	-0.11
Business Intelligence Analyst	1242	0.81	0.88	-0.07
Electronics Engineer	562	0.81	0.86	-0.05
Computer and Information Systems Manager	971	0.80	0.89	-0.09
Other - Military Officer Special and Tactical Operations Leader/Manager	643	0.80	0.72	0.08
Computer Programmer	3194	0.78	0.91	-0.13
Other - Designer	1018	0.77	0.82	-0.05
Information Technology Project Manager	1722	0.77	0.85	-0.08
Civil Engineer	1468	0.76	0.77	-0.01
Risk Management Specialist	514	0.75	0.70	0.05
Supervisor/Manager of Construction and/or Extraction Workers	675	0.75	0.66	0.09
Sales Engineer	585	0.75	0.74	0.01

Table S11: Highest scoring occupations on 3D rotation (in descending order of 3D rotation percentile)

occupation	N	R2D	R3D	Difference
Computer Systems Engineers/Architect	1351	0.89	0.95	-0.06
Computer Software Engineer	4480	0.83	0.93	-0.10
Web Developer	1028	0.84	0.93	-0.09
Engineering Manager	642	0.82	0.93	-0.11
Mechanical Engineer	1034	0.89	0.92	-0.03
Computer Programmer	3194	0.78	0.91	-0.13
Biologists	554	0.90	0.91	-0.01
Computer Security Specialist	581	0.73	0.89	-0.16
Computer and Information Systems Manager	971	0.80	0.89	-0.09
Business Intelligence Analyst	1242	0.81	0.88	-0.07
Network and Computer Systems Administrator	828	0.86	0.87	-0.01
Electrical Engineers	923	0.82	0.86	-0.04
Electronics Engineer	562	0.81	0.86	-0.05
Information Technology Project Manager	1722	0.77	0.85	-0.08
Management Analyst	1360	0.73	0.83	-0.10
Other - Designer	1018	0.77	0.82	-0.05
Architect	1596	0.82	0.81	0.01
Chief Executive Officer	2115	0.70	0.81	-0.11
Instructional Designer and/or Technologist	952	0.65	0.80	-0.15
Computer and Information Scientist	807	0.67	0.79	-0.12

Table S12: Occupations with the greatest difference in performance (in favor of 2D rotation)

occupation	N	R2D	R3D	Difference
Other - Construction and Related Work	555	0.54	0.31	0.23
Emergency Medical Technician and/or Paramedic	573	0.67	0.47	0.20
Medical Records and Health Information Technicians	600	0.33	0.16	0.17
Other - Production Worker	871	0.44	0.27	0.17
Insurance Sales Agent	588	0.28	0.13	0.15
Food Service Attendant/Helper	578	0.33	0.19	0.14
Medical and Clinical Laboratory Technician and/or Technologist	1139	0.44	0.30	0.14
Host/Hostess	866	0.24	0.11	0.13
Supervisor/Manager of Food Preparation and Serving Workers	1202	0.48	0.35	0.13
Supervisor/Manager of Retail Sales Workers	1619	0.44	0.31	0.13
Security Guard	628	0.48	0.36	0.12
Sales Representative - Wholesale and Manufacturing	1264	0.43	0.31	0.12
Pharmacist	1672	0.55	0.44	0.11
Acute Care Nurse	723	0.27	0.17	0.10
Nursing Aid, Orderly and/or Attendant	961	0.23	0.13	0.10

Table S12: Occupations with the greatest difference in performan *(continued)*

occupation	N	R2D	R3D	Difference
Computer Operator	554	0.23	0.13	0.10
Critical Care Nurse	532	0.41	0.31	0.10
Other - Health Technologist and/or Technician	641	0.62	0.52	0.10
Other - Sales Representative	2392	0.24	0.14	0.10
Telemarketer	506	0.20	0.11	0.09

Table S13: Occupations with the greatest difference in performance (in favor of 3D rotation)

occupation	N	R2D	R3D	Difference
Postsecondary Teacher - Psychology	606	0.30	0.74	-0.44
Special Education Teacher - Secondary School	555	0.22	0.50	-0.28
Other - Therapist	1504	0.31	0.57	-0.26
Musician and/or Singer	795	0.55	0.76	-0.21
Librarian	1244	0.49	0.68	-0.19
Budget Analyst	582	0.43	0.62	-0.19
Lawyer	4956	0.58	0.76	-0.18
Copy Writer	946	0.21	0.39	-0.18
Supervisors/Manager of Non-Retail Sales Workers	763	0.31	0.48	-0.17
Computer Security Specialist	581	0.73	0.89	-0.16
Computer Support Specialist	1671	0.63	0.78	-0.15
Instructional Designer and/or Technologist	952	0.65	0.80	-0.15
Writer	1029	0.42	0.57	-0.15
Public Relations Specialist	816	0.29	0.43	-0.14
Psychiatrist	769	0.62	0.76	-0.14
Special Education Teacher - Preschool, Kindergarten, and Elementary School	897	0.21	0.35	-0.14
Counselor - Mental Health	1020	0.34	0.48	-0.14
Art Director	1038	0.47	0.60	-0.13
Computer Programmer	3194	0.78	0.91	-0.13
Editor	1254	0.64	0.76	-0.12

Majors

```r
majors <- ICARdata %>%
  dplyr::group_by(major) %>%
  dplyr::summarise(R2D_Score = mean(R2D_IRT_general, na.rm = TRUE),
  R3D_Score = mean(ICAR_R3D_IRT_score, na.rm = TRUE),
  n = length(!is.na(R2D_IRT_general))) %>%
  dplyr::mutate(R2D_Percentile = round(dplyr::percent_rank(R2D_Score),2),
  R3D_Percentile = round(dplyr::percent_rank(R3D_Score),2) # Percentiles
  )
```

Table S14: Highest scoring majors on 2D rotation (in descending order of 2D rotation percentile)

major	n	R2D	R3D	Difference
Neuroscience	993	1.00	1.00	0.00
Applied Mathematics	1070	0.99	0.99	0.00
Physics	4120	0.99	0.99	0.00
Materials Science and Engineering	1004	0.98	0.97	0.01
Biomedical Engineering	1646	0.97	0.96	0.01
Manufacturing and Design Engineering	1197	0.96	0.73	0.23
Mathematics	4702	0.95	0.95	0.00
Chemical and Biological Engineering	4015	0.94	0.91	0.03
Mechanical Engineering	9727	0.93	0.89	0.04
Aerospace Engineering	1812	0.92	0.90	0.02
Anthropology	1915	0.91	0.92	-0.01
Geological Sciences	1911	0.90	0.84	0.06
Industrial Engineering	2725	0.88	0.88	0.00
Chemistry	5829	0.88	0.87	0.01
Actuarial Sciences	618	0.86	0.92	-0.06
Biology	13885	0.86	0.86	0.00
Other Mathematics Major	619	0.85	0.85	0.00
Religion	803	0.83	0.84	-0.01

Table S14: Highest scoring majors on 2D rotati *(continued)*

major	n	R2D	R3D	Difference
Electrical Engineering	10574	0.82	0.81	0.01
Philosophy	2202	0.81	0.95	-0.14

Table S15: Highest scoring majors on 3D rotation (in descending order of 3D rotation percentile)

major	n	R2D	R3D	Difference
Neuroscience	993	1.00	1.00	0.00
Applied Mathematics	1070	0.99	0.99	0.00
Physics	4120	0.99	0.99	0.00
Materials Science and Engineering	1004	0.98	0.97	0.01
Biomedical Engineering	1646	0.97	0.96	0.01
Mathematics	4702	0.95	0.95	0.00
Philosophy	2202	0.81	0.95	-0.14
Actuarial Sciences	618	0.86	0.92	-0.06
Anthropology	1915	0.91	0.92	-0.01
Chemical and Biological Engineering	4015	0.94	0.91	0.03
Aerospace Engineering	1812	0.92	0.90	0.02
Statistics	1428	0.77	0.90	-0.13
Mechanical Engineering	9727	0.93	0.89	0.04
Music	3066	0.79	0.88	-0.09
Industrial Engineering	2725	0.88	0.88	0.00
Chemistry	5829	0.88	0.87	0.01
Biology	13885	0.86	0.86	0.00
Other Mathematics Major	619	0.85	0.85	0.00
Geological Sciences	1911	0.90	0.84	0.06
Religion	803	0.83	0.84	-0.01

Table S16: Majors with the greatest discrepancy in performance (in favor of 2D rotation)

major	n	R2D	R3D	Difference
Culinary Arts and Sciences	605	0.45	0.15	0.30
Medical Laboratory/Technology	4117	0.37	0.07	0.30
Fashion	2010	0.34	0.05	0.29
Pharmacology	4866	0.49	0.23	0.26
Criminal Justice and Corrections	2412	0.28	0.04	0.24
Manufacturing and Design Engineering	1197	0.96	0.73	0.23
Science Education	2412	0.36	0.14	0.22
Animal Sciences	2335	0.60	0.41	0.19
Other Natural Sciences Major	6196	0.71	0.54	0.17
Interior Design	2090	0.61	0.45	0.16
Nutrition and Wellness	2265	0.40	0.25	0.15
Accounting	18603	0.41	0.26	0.15
Botany	1543	0.43	0.29	0.14
Other Medicine and Allied Health Major	15935	0.42	0.29	0.13
Dentistry	4441	0.22	0.10	0.12
Graphic Arts	4478	0.69	0.58	0.11
Mathematics Education	1072	0.78	0.67	0.11
Other Engineering and Technology Major	11620	0.75	0.64	0.11
Fiction Writing	752	0.80	0.70	0.10
Nursing	16436	0.18	0.08	0.10

Table S17: Majors with the greatest discrepancy in performance (in favor of 3D rotation)

major	n	R2D	R3D	Difference
Special Education	2030	0.18	0.40	-0.22
Communication Sciences	1148	0.21	0.43	-0.22

Table S17: Majors with the greatest discrepancy in performan *(continued)*

major	n	R2D	R3D	Difference
Journalism	3806	0.15	0.36	-0.21
Radio, Television and Film Communication	1732	0.32	0.49	-0.17
Spanish	765	0.50	0.66	-0.16
Government	1198	0.23	0.39	-0.16
Law and Legal Studies	8363	0.27	0.42	-0.15
Public Relations and Advertising	2909	0.10	0.24	-0.14
Philosophy	2202	0.81	0.95	-0.14
Statistics	1428	0.77	0.90	-0.13
Computer Programming	14379	0.68	0.80	-0.12
Management Information Systems	1844	0.65	0.77	-0.12
Economics	5976	0.70	0.82	-0.12
International Studies	750	0.57	0.68	-0.11
Linguistics	1783	0.68	0.79	-0.11
Political Science	9189	0.46	0.56	-0.10
Other Communications Major	2112	0.25	0.34	-0.09
Other Cultural and Regional Studies Major	987	0.36	0.45	-0.09
Music	3066	0.79	0.88	-0.09
History	4872	0.52	0.61	-0.09
